# DNA polymerase POLD1 promotes proliferation and metastasis of bladder cancer by stabilizing MYC

**DOI:** 10.1038/s41467-023-38160-x

**Published:** 2023-04-27

**Authors:** Yejinpeng Wang, Lingao Ju, Gang Wang, Kaiyu Qian, Wan Jin, Mingxing Li, Jingtian Yu, Yiliang Shi, Yongzhi Wang, Yi Zhang, Yu Xiao, Xinghuan Wang

**Affiliations:** 1 Department of Urology, Zhongnan Hospital of Wuhan University, Wuhan, China; 2 Department of Biological Repositories, Zhongnan Hospital of Wuhan University, Wuhan, China; 3 Human Genetic Resources Preservation Center of Hubei Province, Wuhan, China; 4 Wuhan Research Center for Infectious Diseases and Cancer, Chinese Academy of Medical Sciences, Wuhan, China; 5 Euler Technology, ZGC Life Sciences Park, Beijing, China; 6 Center for Quantitative Biology, School of Life Sciences, Peking University, Beijing, China; 7 Medical Research Institute, Wuhan University, Wuhan, China; 8 Institute of Urology, Wuhan University, Wuhan, China

**Keywords:** Bladder cancer, Ubiquitylation, Oncogenes

## Abstract

To date, most studies on the DNA polymerase, POLD1, have focused on the effect of POLD1 inactivation mutations in tumors. However, the implications of high POLD1 expression in tumorigenesis remains elusive. Here, we determine that POLD1 has a pro-carcinogenic role in bladder cancer (BLCA) and is associated to the malignancy and prognosis of BLCA. Our studies demonstrate that POLD1 promotes the proliferation and metastasis of BLCA via MYC. Mechanistically, POLD1 stabilizes MYC in a manner independent of its’ DNA polymerase activity. Instead, POLD1 attenuates FBXW7-mediated ubiquitination degradation of MYC by directly binding to the MYC homology box 1 domain competitively with FBXW7. Moreover, we find that POLD1 forms a complex with MYC to promote the transcriptional activity of MYC. In turn, MYC increases expression of *POLD1*, forming a POLD1-MYC positive feedback loop to enhance the pro-carcinogenic effect of POLD1-MYC on BLCA. Overall, our study identifies POLD1 as a promotor of BCLA via a MYC driven mechanism and suggest its potential as biomarker for BLCA.

## Abstract (web summary)

DNA polymerase 1 (POLD1) has an important role in DNA damage repair and is frequently upregulated in cancer. Here, the authors report a non-canonical role of POLD1 wherein it stabilises MYC protein, creating a positive feedback loop with POLD1 expression and driving bladder cancer progression and metastasis.

## Introduction

Bladder cancer (BLCA) is well established as one of the most common malignancies of the urinary system, accounting for approximately 570,000 new cases and 210,000 deaths worldwide each year^1^. It is one of the cancers with the highest economic burden because of its propensity to relapse and the necessity for ongoing monitoring and follow-up^2^. Accordingly, it is essential to explore the mechanisms that underlie the occurrence and development of BLCA to find a diagnosis and treatment method.

Our research group has focused on exploring the mechanism of BLCA tumorigenesis^3–5^. We discovered that DNA polymerase (POLD1) pedigree mutations might be a risk factor for BLCA and that patients with *POLD1*^*G178R*^ mutations tended to have higher tumor mutation burden (TMB), immune cell invasion, and longer survival times than patients with *POLD1* wild-type BLCA^6^. Hence, in BLCA, we further explored its molecular mechanism. *POLD1* encodes p125, the primary catalytic subunit of Pol δ^7^. p125 consists of two major domains: a catalytic core N-terminal domain with polymerase and exonuclease activities and a metal-binding C-terminal domain. The C-terminal domain of the POLD1 has a proliferating cell nuclear antigen (PCNA) interaction motif between amino acids 1001 and 1005, which is essential for POLD1 enzyme activity^8,9^. Current studies of POLD1 focus on its hypermutation’s effects on tumor immune microenvironments, and it has the potential as a biomarker to track the effectiveness of immunotherapy^6,10,11^. Pathogenic *POLD1* exonuclease domain mutations might impair polymerase’s capacity to proofread, and a wealth of data suggests that the *POLD1* pedigree mutation may be a predisposition factor for colorectal cancer^12–14^. Besides that, it was discovered that tumors with DNA polymerase epsilon (*POLE*)/*POLD1* mutations had higher proportions of CD8^+^ tumor-infiltrating lymphocytes, indicating that patients with this kind may benefit more from immune checkpoint inhibitor therapy. Moreover, patients with mutations in the proofreading domain had better predictive outcomes^15^, which is consistent with our earlier findings^6^. Substantial evidence suggests that POLD1 is involved in DNA damage repair, cell cycle progression, cell aging, and other regulatory pathways, influencing the occurrence and development of various tumors^16–18^. Current evidence suggests that liver cancer patients with high levels of POLD1 tend to form an inhibitory tumor microenvironment and tend to lead to tumor progression^19^. In breast cancer, the high expression of POLD1 is closely related to poor prognosis. Knocking down *POLD1* can inhibit the proliferation of breast cancer cells and lead to cell cycle disorders^20^. In addition, SIRT1 can affect the invasion and migration of breast cancer cells by upregulating POLD1 expression^21^. However, the exact way that high levels of POLD1 cause cancer is still unknown.

MYC is a transcription factor that has been shown to be involved in a wide range of biological processes, including the cell proliferation and differentiation, epithelial to mesenchymal transition (EMT), genomic instability, tumor development, tumor microenvironment modulation, as well as many other processes^22–29^. As a fragile protein with a brief half-life, MYC is primarily degraded by the ubiquitin-proteasome pathway. Degradation of MYC involves a cascade of phosphorylation at serine 62 (S62) phosphorylated by ERK/CDK and threonine 58 (T58) regulated by GSK3β^30,31^. After that, MYC phosphorylated at T58 is recognized by the E3 ubiquitin ligase FBXW7 and subsequently degraded by the 26S proteasome^32–34^. In addition, the degradation process of MYC is also mediated by other molecules. For instance, TRIB3 encourages the growth of lymphoma by inhibiting the interaction between MYC and the E3 ligase UBE3B^35^. AURKB competitively binds MYC with the E3 ligase FBXW7 to increase the stability of the MYC protein, which contributes to the development of T-cell leukemia^36^. In addition, *MYC* knockdown in the BLCA cell lines T24 and 5637 reportedly inhibits the ability of proliferation and metastasis of BLCA^37^. However, the specific mechanism underlying the regulatory role of MYC in the occurrence and development of BLCA remains unclear.

In this work, we report POLD1 stabilization of MYC via competitive binding with FBXW7, resulting in decreased ubiquitination-mediated degradation of MYC. Subsequently, elevated levels of MYC increase the transcription of *POLD1*, forming a positive feedback loop and accelerating BLCA proliferation and metastasis.

## Results

### POLD1 is correlated with the malignancy and prognosis of BLCA

**Fig. 1:**
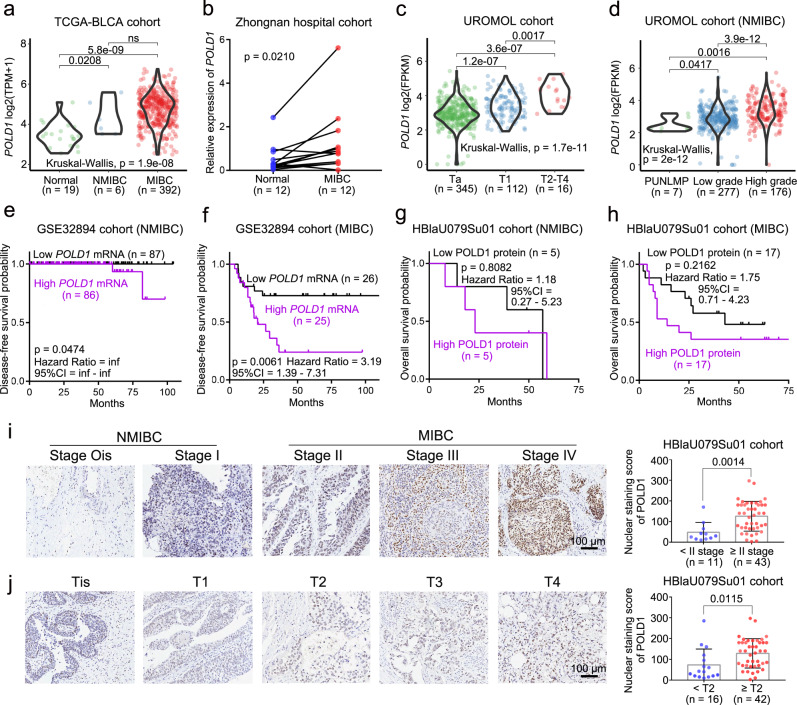
The expression of POLD1 was correlated with the malignant degree and prognosis of BLCA. The mRNA level of *POLD1* in BLCA (MIBC or NMIBC) and normal tissues in **a** TCGA-BLCA (RNA-seq data) and **b** Zhongnan Hospital cohort (RT-qPCR data). Statistical significance was determined by two-tailed Wilcoxon rank-sum test (**a**) and paired two-tailed Student’s *T*-test (**b**). The mRNA level of *POLD1* in different **c** stages (Ta, T1, T2, T3, and T4) and **d** pathology grades: Papillary Urothelial Neoplasms of Low Malignant Potential (PUNLMP), low grade and high grade in the UROMOL^39^ cohort. Statistical significance was determined by two-tailed Wilcoxon rank-sum test and Kruskal–Wallis test (**c**, **d**). The prognostic curve (disease-free survival) of different *POLD1* mRNA levels in the two subtypes of **e** NMIBC and **f** MIBC in the GSE32894^40^ cohort was analyzed. The patients were divided into a high *POLD1* mRNA level group and a low *POLD1* mRNA level group according to the median *POLD1* expression. The prognostic curve (overall survival) of different POLD1 protein levels in the two subtypes of **g** NMIBC and **h** MIBC in the HBlaU079Su01 cohort was analyzed. The patients were divided into high and low POLD1 protein level groups according to the median POLD1 protein level. Statistical significance was determined by the log-rank test of Kaplan–Meier analysis (**e**–**h**). The purple line represents the group with high *POLD1* mRNA or protein expression and the black line represents the group with low *POLD1* mRNA or protein expression. Representative pictures (left panel) and statistical figures (right panel) of immunohistochemistry staining analysis of POLD1 protein levels in different **i** stages (The seventh edition of AJCC: stage Ois, stage I, stage II, stage III, and stage IV), and **j** T stages (Tis, T1, T2, T3, and T4) in the HBlaU079Su01 cohort. POLD1 nuclear staining score = The intensity of nuclear staining × Staining positive area. Data are mean ± SD. Statistical significance was determined by the two-tailed Student’s *T*-test (**i**, **j**). The *n* number represents *n* biologically independent patient samples in each group. Exact *n* values are marked in the images. NMIBC non-muscle-invasive bladder cancer, MIBC muscle-invasive bladder cancer, CI confidence interval, inf infinite, TPM transcripts per kilobase million, FPKM fragments per kilobase million. Source data are provided as a Source Data file; ns: not significant.

Like in most malignancies, *POLD1* is highly expressed in BLCA. Amplification may be one of the reasons for *POLD1* upregulation in most tumors (Supplementary Fig. 1a, b). In particular, we found that several other members of DNA polymerase δ, including *POLD2*, *POLD3*, and *POLD4*, were not significantly upregulated in BLCA (Supplementary Fig. 1c), which implies that *POLD1* may act in BLCA in a manner that is independent of DNA polymerase. According to the degree of local tumor invasion, BLCA is currently separated clinically into muscle-invasive bladder cancer (MIBC) and non-muscle-invasive bladder cancer (NMIBC). We gathered a lot of information to look at the connection between *POLD1* expression and clinical data on BLCA. The *POLD1* mRNA expression level was notably higher in NMIBC or MIBC tissues compared to normal adjacent tissues in the TCGA-BLCA (normal: *n* = 19, NMIBC: *n* = 6, MIBC: *n* = 392) and Zhongnan Hospital (normal: *n* = 12, MIBC: *n* = 12) cohort studies (Fig. 1a, b). In the GSE13507^38^ cohort (NMIBC: *n* = 103, MIBC = 62), we found that *POLD1* levels were notably higher in MIBC than in NMIBC (Supplementary Fig. 1d). Additionally, *POLD1* was significantly positively correlated with tumor T stage (Ta: *n* = 345, T1: *n* = 112, T2-T4: *n* = 16), pathological grade (PUNLMP (papillary urothelial neoplasms of low malignant potential): *n* = 7, low grade: *n* = 277, high grade: *n* = 176) and tumor size (<3 cm: *n* = 283, ≥3 cm: *n* = 87) in the UROMOL^39^ cohort (Fig. 1c, d and Supplementary Fig. 1e). In terms of prognosis, we found that the transcription level of *POLD1* was significantly correlated with the disease-free survival of NMIBC (HR (hazard ratio) = inf (infinity), 95% CI (confidence interval) = inf - inf, *p* = 0.0474) and MIBC (HR = 3.19, 95% CI = 1.39–7.31, *p* = 0.0061) in the GSE32894^40^ cohort (Fig. 1e, f). In the UROMOL cohort, *POLD1* exhibited a significant correlation with progression-free survival of NMIBC (HR = 4.47, 95% CI = 2.21–9.05, *p* = 0.0002, Supplementary Fig. 2a), and no progressive events were observed in patients with MIBC, hence no meaningful results could be made (Supplementary Fig. 2b). Even though there was no statistically significant difference, patients with different POLD1 protein levels in our HBLaU079Su01 cohort (NMIBC: HR = 1.18, 95% CI = 0.27–5.23, *p* = 0.8082; MIBC: HR = 1.75, 95% CI = 0.71–4.23, *p* = 0.2162) also demonstrated a trend that was in line with the previous conclusions (Fig. 1g, h). Similarly, patients with high *POLD1* expression in the GSE13507^38^ cohort tended to have worse progression-free survival outcomes (NMIBC: HR = 2.07, 95% CI = 0.63–6.77, *p* = 0.2146; MIBC: HR = 1.21, 95% CI = 0.50–2.90, *p* = 0.6719), even if there was no significant difference (Supplementary Fig. 2c, d). In addition, in our HBLaU079Su01 cohort, we found that the protein level of POLD1 was also positively correlated with the stage (ATCC stage: Ois, I, II, III, and IV) and T stage (Tis, T1, T2, T3, and T4) of BLCA (Fig. 1i, j) and was notably increased in normal tissues, NMIBC, and MIBC in turn (Supplementary Fig. 2e). These results suggest that POLD1 may play a role in the tumorigenesis and progression of BLCA.

### POLD1 promotes BLCA proliferation and metastasis in vivo and in vitro

**Fig. 2:**
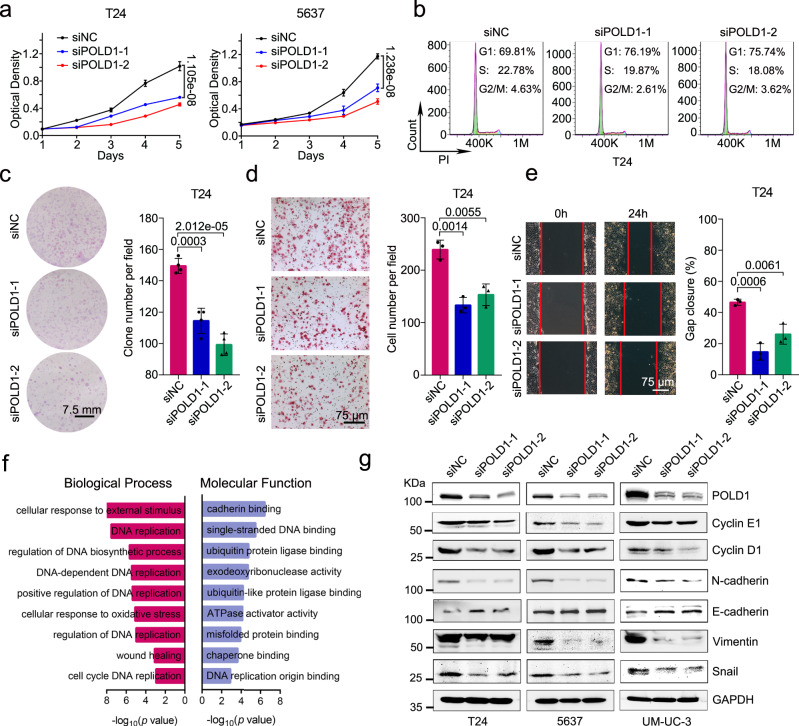
POLD1 promotes BLCA proliferation and metastasis in vitro. **a** The cell proliferation curve of T24 and 5637 cells with *POLD1* knockdown. Before detecting the absorbance, 20 μL of MTT was added to the 96-well seeded with cells, and incubated in a 37°C cell incubator for 4 h. Then, 200 μL of DMSO was added to each well and shaken until homogenized. Graph shows mean ± SD, *n* = 6 biologically independent experiments in each group. **b** Flow cytometric analysis of DNA content for cell cycle progression from propidium iodide (PI, 100 μg/mL) staining of T24 cells with or without *POLD1* depletion. **c** Representative images (left panel) and statistical graph (right panel) of clone formation assays from the indicated groups with *POLD1* depletion in T24 cells (*n* = 4 biologically independent experiments in each group). **d** Representative images (left panel) and cell numbers (right panel) of Transwell assays from the indicated groups with or without *POLD1* knockdown in T24 cells (*n* = 3 biologically independent experiments in each group). **e** Representative images (left panel) and statistical results (right panel) of the wound healing assay in T24 cells with or without *POLD1* depletion (*n* = 3 biologically independent experiments in each group). Gap closure (%) = (0 h distance − 24 h distance)/0 h distance 100%. The “distance” here refers to the shortest distance between the red lines in the diagram. **f** Gene Ontology (GO) biological processes (red) and molecular functions (blue) related to POLD1 from enrichment analysis of differentially expressed genes (DEGs) from RNA-seq assays. The GO analysis here was performed by the R package “clusterProfiler”. *p* values computed using two-tailed Fisher’s exact test with *p* value <0.05 were used as the threshold for statistical tests. **g** Western blot analyses of cell cycle- and EMT-related proteins with or without *POLD1* knockdown in T24, 5637, and UM-UC-3 cells. GAPDH was used as the loading control. Statistical significance was determined by two-tailed Student’s *T*-test (**a**, **b**, **d**, **e**). Source data are provided as a Source Data file.

To examine the function of POLD1 in regulating the phenotype of BLCA, we constructed three *POLD1* siRNA and POLD1 overexpressing plasmids. We confirmed their knockdown or overexpression efficiency by RT-qPCR and selected the two siRNAs (*siPOLD1-1* and *siPOLD1-2*) with the highest efficiency for subsequent experiments (Supplementary Fig. 3a). The MTT assay showed that the proliferation of T24 and 5637 cells was significantly inhibited and enhanced by knockdown and overexpression of *POLD1*, respectively (Fig. 2a and Supplementary Fig. 3b). Knockdown of *POLD1* in T24 (Fig. 2b) and 5637 (Supplementary Fig. 3c) cells resulted in cell cycle arrest at the G1 phase. The colony formation assay results were consistent with those of MTT assays (Fig. 2c and Supplementary Fig. 3d, e). In addition, the transwell assays (Fig. 2d and Supplementary Fig. 3f) and wound healing assays (Fig. 2e and Supplementary Fig. 3g) revealed that the migration ability of T24 and 5637 cells was significantly weakened after *POLD1* suppression. In contrast, overexpression of POLD1 enhanced the migration ability of T24 and 5637 cells (Supplementary Fig. 3h). We performed an RNA-seq assay after *POLD1* knockdown in 5637 cells, and then functional annotation (GSEA and GO) analysis showed that *POLD1* was significantly enriched in pathways involving the cell cycle, DNA replication, wound healing, cadherin binding, and EMT-related signals (Fig. 2f, Supplementary Fig. 4a, and Supplementary Tables 1, 2). Subsequently, immunoblotting was used to detect the expression of cell cycle and EMT-related proteins. After *POLD1* downregulation in T24, 5637, and UM-UC-3 cell lines, we observed that the Cyclin E1, Cyclin D1, N-cadherin, Vimentin, and Snail protein levels were considerably lowered, whereas E-cadherin levels were elevated (Fig. 2g). With POLD1 overexpression, the opposite outcomes were seen (Supplementary Fig. 4b). Immunofluorescence staining of *POLD1*-downregulated 5637 cells showed that the fluorescence levels of Cyclin E1, Ki67 (a marker of a cell’s ability to proliferate), and N-cadherin decreased significantly (Supplementary Fig. 4c). Thus, the above results indicate that POLD1 encourages the proliferation and metastasis of BLCA by promoting G1-S phase transformation and the EMT process.

**Fig. 3:**
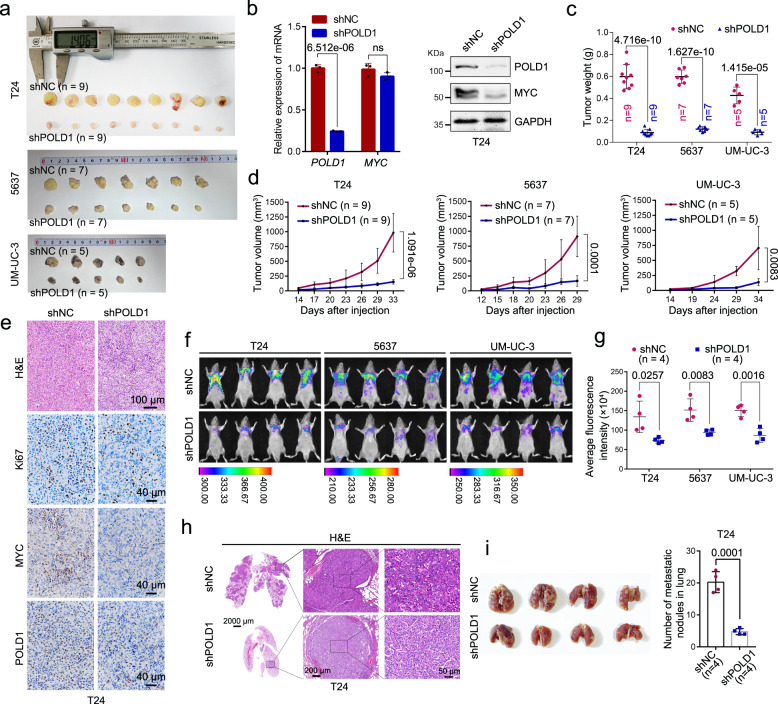
POLD1 promotes BLCA proliferation and metastasis in vivo. **a** Gross view of a subcutaneous tumor derived from a xenograft model (*shNC* vs. *shPODL1*) using different BLCA cells (T24: *n* = 9 per group; 5637: *n* = 7 per group; UM-UC-3: *n* = 5 per group). **b** Validation of the knockdown efficiency of T24 stable cell lines at the transcriptional (by qPCR assay, left panel). Graph shows mean ± SD, *n* = 3 biologically independent experiments in each group. And protein levels (by Western blot assay, right panel). Effects of *POLD1* knockdown on tumor **c** weight and **d** volume in tumor-bearing mice models (T24: *n* = 9 per group; 5637: *n* = 7 per group; UM-UC-3: *n* = 5 per group). **e** Immunohistochemical (IHC) and H&E staining analysis of tumor tissue in subcutaneous tumor-bearing model of T24 stable *shNC* and *shPOLD1* cells. Ki67 was used as a proliferative marker to reflect the proliferative ability of the tumor. **f** The stable cell lines (T24: *n* = 4 per group, 5637: *n* = 4 per group, UM-UC-3: *n* = 4 per group) of *shNC* or *shPOLD1* were injected into mice through the tail vein, and GFP fluorescence intensity in mice was detected 8 weeks later. **g** Statistical diagram of average GFP fluorescence intensity in the lung region of nude mice (T24: *n* = 4 per group, 5637: *n* = 4 per group, UM-UC-3: *n* = 4 per group). The fluorescence intensity of GFP was evaluated by ImageJ software. Statistical significance was determined by two-tailed Student’s *T*-test. No adjustments were made for multiple comparisons. **h** Representative images of H&E-stained mice lung sections showed tumor lesions in lung tissue 8 weeks after *shNC* or *shPOLD1* T24 cell tail vein injection (*n* = 4 mice per group). **i** Gross view of the lung in the tail vein injection lung metastasis model (left panel). Statistical analysis of the number of tumors on the lung surface (right panel, *n* = 4 mice per group). For Western blot experiments, GAPDH was used as the loading control (**b**). Statistical significance was determined by two-tailed Student’s *T*-test (**b**–**d**, **f**, **g**, **i**). ns not significant. Source data are provided as a Source Data file.

To investigate the role of POLD1 in the carcinogenesis and metastasis of BLCA in vivo, we established a tumor-bearing model by subcutaneous injection and a lung metastasis model by tail vein injection in NOID/SCID mice. We generated *shNC* and *shPOLD1* stable cell lines in T24, 5637, and UM-UC-3 cells and conducted subcutaneous tumor-bearing experiments after the transcriptional and protein levels verified their knockdown efficiency. The collected subcutaneous tumor tissues exhibited that the tumor size in the *shPOLD1* group was much less than that of the *shNC* group (Fig. 3a, b). The results in the xenograft model confirmed that in vivo depletion of *POLD1* notably reduced the weight (Fig. 3c) and proliferation rate (Fig. 3d) of subcutaneous tumors. Immunohistochemical (IHC) staining demonstrated that the abundance of POLD1 and Ki67 decreased significantly after *POLD1* depletion, which was consistent with earlier in vitro findings (Fig. 3e). After tail vein injection in mice, the number and size of lung metastasis foci with *POLD1* depletion were notably reduced compared to the *shNC* group, and GFP fluorescence was relatively weaker (Fig. 3f–i). The above evidence suggests that POLD1 can promote the proliferation and metastasis of BLCA in vitro and in vivo.

### Depletion of *POLD1* weakened the stability of MYC

**Fig. 4:**
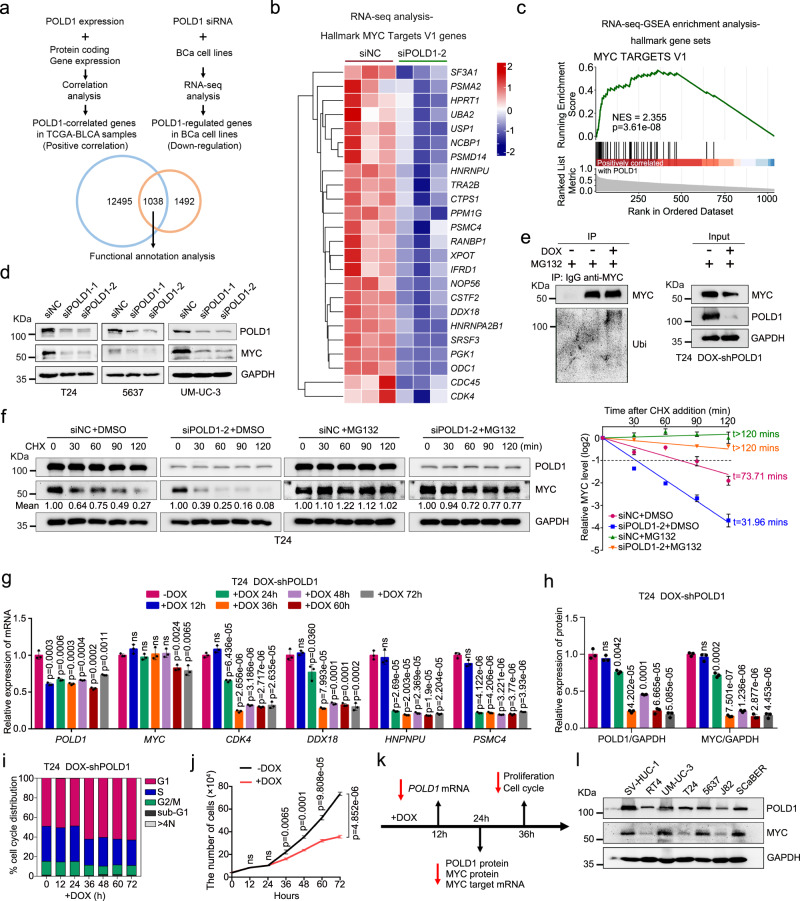
*POLD1* depletion weakened the protein stability of MYC. **a** Schematic of the identification of *POLD1* positively correlated genes in the TCGA-BLCA database (the blue circle, statistical significance was determined by Jarque-Bera test) and genes that may be positively regulated by POLD1 in 5637 cells (the brown circle, statistical significance was determined by two-tailed Wald test). **b** A Heatmap of genes with significant changes in the Hallmark_MYC_Targets_V1 gene set upon *POLD1* knockdown in 5637 cells (*n* = 3). Statistical significance was determined by two-tailed Wald test. **c** POLD1 related gene set enrichment analysis (GSEA). Statistical significance was determined by two-tailed Fisher’s exact test. **d** After *POLD1* knockdown in T24, 5637, and UM-UC-3 cells with specific siRNA, Western blot analysis showed that the protein levels of MYC were significantly reduced. **e** Western blot analysis of endogenous ubiquitination after MYC-IP under denaturing conditions in DOX-inducible *shPOLD1* T24 cells. **f** Representative images of Western blot analysis of the effect of *POLD1* depletion on MYC degradation in T24 cells incubated with CHX (50 μg/mL) or CHX plus MG132 (10 μM) for the indicated times (left panel) and statistical diagram of protein half-life assays (right panel, *n* = 3 biologically independent experiments in each group). **g** qPCR analysis of *MYC* and its target genes in DOX-induced *shPOLD1* T24 cells (Graph shows mean ± SD, *n* = 3 biologically independent experiments in each group). **h** Representative images of Western blot analysis of the expression changes of POLD1 and MYC after *POLD1* knockdown induced by DOX at gradient time point (*n* = 3 biologically independent experiments in each group). The protein levels of POLD1 and MYC were quantified by ImageJ software and standardized to GAPDH. **i** Cell cycle and **j** proliferation analysis after *POLD1* knockdown induced by DOX at gradient time points (Graph shows mean ± SD, n = 3 biologically independent experiments in each group). **k** Timeline of all assay results of DOX-induced *POLD1* knockdown in this section. **l** Western blot analysis of POLD1 and MYC expression in different BLCA cell lines. For Western blot experiments, GAPDH was used as a loading control (**d**–**f**, **h**, **l**). Statistical significance was determined by two-tailed Student’s *T*-test (**g**, **h**, **j**). NES: Normalized Enrichment Score; ns: not significant. Source data are provided as a Source Data file.

To understand the mechanism of the downstream effect of POLD1 on BLCA, we referred to Wang et al.’s bioinformatics analysis strategy^41^. We performed RNA-seq analysis after *POLD1* knockdown in 5637 cells. Since the results produced by using only a single siRNA may have off-target effects, we only focused on genes with the same regulatory direction in subsequent analyses, namely, genes that were positively correlated with *POLD1* in TCGA-BCLA and genes that were significantly downregulated after *POLD1* knockdown. Finally, we obtained 1038 genes as candidate genes for subsequent analysis (Fig. 4a and Supplementary Dataset 1). The GSEA results showed that the enrichment score of MYC-targeted signaling pathways was significantly reduced after *POLD1* knockdown (Fig. 4b, c and Supplementary Table 1), indicating that POLD1 may regulate MYC-related downstream signaling. Not surprisingly, the changes in MYC target genes in 5637 cells after *POLD1* knockdown by qPCR were roughly consistent with the results of RNA-seq (Supplementary Fig. 5a). It should be noted that the *MYC* transcription level was not notably affected after *POLD1* knockdown (Supplementary Fig. 5a), which is consistent with the findings in stable cell lines (Fig. 3b). Furthermore, by Western blot and immunofluorescence assays, we detected that weakened or enhanced *POLD1* expression in T24, 5637, and UM-UC-3 cells resulted in a significant decrease or increase in MYC abundance (Fig. 4d and Supplementary Fig. 4b, c). IHC assays in vivo also exhibited a consistent trend (Fig. 3e). Altogether, the above results indicate that POLD1 might affect the posttranslational modification process of MYC. An increasing body of evidence suggests that MYC is degraded mainly by the ubiquitin-proteasome pathway^32–34^. We investigated whether POLD1 could regulate the degradation process of MYC. The results showed that MG132 (a specific 26S proteasome inhibitor) could notably rescued the decrease in MYC abundance caused by *POLD1* depletion (Supplementary Fig. 5b). Furthermore, we found that *POLD1* knockdown induced by doxycycline (DOX) increased the endogenous level of MYC ubiquitination in T24 cells (Fig. 4e). Notably, after *POLD1* was knocked down in T24 cells, we detected a significant acceleration of MYC degradation in the half-life assay, and this impact was almost completely rescued by administration of MG132 (Fig. 4f). These findings imply that POLD1 stabilizes MYC by mediating its degradation through the proteasome pathway.

To determine whether POLD1 promotes the proliferation and metastasis of BLCA by stabilizing MYC, we performed a series of rescue assays. Immunoblot assays confirmed that overexpression of MYC could effectively prevent *POLD1* downregulation from affecting Cyclin E1, Cyclin D1, N-cadherin, and Snail expression (Supplementary Fig. 5c). MTT and transwell assays showed that overexpression of MYC significantly reversed the *POLD1* depletion-induced decline in proliferation and migration capacity in T24 and 5637 cells (Supplementary Fig. 5d–f).

Since *POLD1* knockdown causes a strong effect on proliferation and the cell cycle, MYC downregulation might be a reaction to these changes. To better understand the time window of POLD1 regulation of MYC, we generated DOX-inducible *POLD1* knockdown T24 cell lines. After DOX induction at different time points, we clarified whether POLD1 regulation of MYC depended on phenotypic changes according to the sequence of phenotypic changes and MYC depletion. At the transcriptional level, *POLD1* was significantly inhibited after DOX treatment for 12 h, while *MYC* exhibited a minimal effect (Fig. 4g). At the protein level, POLD1 and MYC were notably decreased almost simultaneously after DOX treatment for 24 h (Fig. 4h and Supplementary Fig. 5g). It should be noted that MYC target genes (*CDK4*, *DDX18*, *HNPRPU*, and *PSMC4*, which were also differentially expressed genes in the previous RNA-seq analysis. Figure 4b and Supplementary Fig. 5a) were significantly downregulated after 24 h of administration of DOX (Fig. 4g), which may be due to MYC loss during this time period (Fig. 4h and Supplementary Fig. 5g). In terms of phenotypes, we found that cell cycle aberration and significant inhibition of the proliferative ability of T24 cells appeared after 36 h of DOX administration (Fig. 4i, j), which obviously occurred after the change in MYC protein levels (Fig. 4k). Taken together, we argue that the effect of POLD1 on the phenotype exhibits a minimal association with the stability of POLD1-regulated MYC.

To understand variations in POLD1 and MYC expression during the cell cycle, we conducted a cycle synchronization assay. The results showed that POLD1 mainly accumulated from the S phase to the peak and gradually decreased until the next S phase (Supplementary Fig. 5h). Interestingly, the cyclic variation in MYC expression was consistent with POLD1 (Supplementary Fig. 5h). As expected, POLD1 and MYC expression variations were consistent with Cyclin D1 and Cyclin E1, which may play essential roles in the G1-S phase^42–44^ (Supplementary Fig. 5h). As expected, we also found consistency between POLD1 and MYC expression in 7 bladder cell lines (SV-HUC-1, RT4, UM-UC-3, T24, 5637, J82, and SCaBER) of BLCA (Fig. 4l).

These data highlight the specific role of POLD1 in MYC stabilization and the fact that POLD1 regulates MYC within a narrow time window. Notably, this regulatory relationship has a minimal association with a notably change in phenotype caused by POLD1.

### POLD1 is a binding partner of MYC

**Fig. 5:**
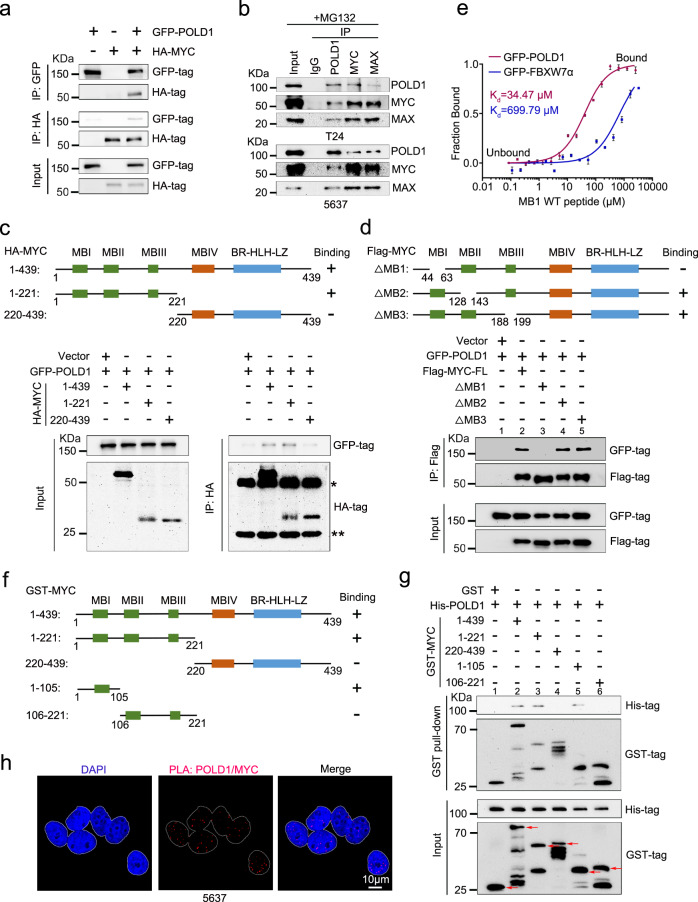
POLD1 is a direct binding partner of MYC. **a** Western blot analysis of GFP-POLD1 and HA-MYC after GFP-IP or HA-IP in 293 T cells. The input was 10% of the extract used for the IP. **b** Western blot analysis of POLD1, MYC, and MAX after IgG, POLD1-IP, MYC-IP, or MAX-IP in T24 (top panel) and 5637 (bottom panel) cells. MAX was used as a positive control for MYC endogenous interaction. **c** Schematic diagram of various MYC truncations in the Co-IP assays (top panel). Western blot analysis of GFP-POLD1 and HA-MYC after HA-IP in 293T cells (bottom panel). **d** Schematic diagram of various MYC deletion mutations in POLD1 binding assays (top panel). Western blot analysis of GFP-POLD1 and Flag-MYC after Flag-IP in 293T cells (bottom panel). **e** MST analysis was used to measure the binding affinity of lysates of overexpressed GFP-POLD1 in 293T cells and the MYC-MB1 (WT) peptide (Graph shows mean ± SD from three biologically independent experiments in each group). Here, GFP-FBXW7α was used as a positive control. The centerline indicates the median, bounds of box = 25th and 75th percentiles, bars = 10th and 90th percentiles, whiskers = min to max. **f** Schematic diagram of various recombinant full-length and fragment GST-MYC proteins in the GST pull-down assays (left panel). **g** Western blot analysis of His-POLD1, GST-MYC after GST pull-down assays (right panel). The red arrows indicate the theoretical location of the full-length and fragment GST-MYC or GST. **h** Confocal microscopy images of PLA of the POLD1 and MYC interaction in 5637 cells. Data are representative images from three independent assays. MBI MYC homology box 1, MBII MYC homology box 2, MBIII MYC homology box 3, MBIV MYC homology box 4, BR-HLH-LZ basic region (BR), and helix-loop-helix-leucine zipper (HLH-LZ) domain, ΔMB MYC box deletion mutants; * represents the heavy chain and ** represents the light chain. Source data are provided as a Source Data file.

By using mass spectrometry, Koch et al.^45^ reported a potential connection between POLD1 and MYC. To verify their association, we performed a number of Co-immunoprecipitation tests (Co-IP). We overexpressed GFP-POLD1 and HA-MYC in 293T cells and demonstrated the interaction between them by Co-IP assay (Fig. 5a). Moreover, we detected an endogenous interaction between POLD1 and MYC in T24 and 5637 cells, and MAX (also known as MYC-associated factor X) was used as a positive control for interaction with MYC (Fig. 5b). To further determine the precise binding region of POLD1 and MYC, we enforced the expression of full-length GFP-POLD1 and fragmented HA-MYC in 293T cells. Through a Co-IP assay, we found that POLD1 bound to the N-terminal (amino acids 1-221) domain of MYC (Fig. 5c). Furthermore, we found that the interaction between GFP-POLD1 and Flag-MYC lacking the homology box 1 (MB1) domain^33^ (the region of interaction with FBXW7α, and also the region of the S62 and T58 phosphorylation sites) was notably lost (Fig. 5d, compare lane 3 vs lane 2). In addition, after the overexpression of GFP-POLD1 in 293T cells, we detected an affinity between POLD1 and MB1 peptide by MST assay, and FBXW7α was used as a positive control. Notably, we found that the affinity of POLD1 for MB1 was stronger than that of FBXW7α (Fig. 5e). To test whether POLD1 has a direct interaction with MYC, we generated a fragmented GST-MYC purified protein to perform a GST pull-down assay with His-POLD1. As expected, the results showed that POLD1 could directly bind to the MB1 domain of MYC (Fig. 5f, g, lane 5).

To determine the specific location of MYC binding on POLD1, we enhanced the expression of the N-terminal (amino acids 1-984) and C-terminal (amino acids 985-1107) of GFP-POLD1 and full-length HA-MYC in 293T cells. Through Co-IP assays, we found that MYC could bind not only to the N-terminal of POLD1 but also to the C-terminal (Supplementary Fig. 6). We further demonstrated colocalization between POLD1 and MYC by PLA in 5637 cells (Fig. 5h). Altogether, these results demonstrate a direct interaction between POLD1 and MYC.

### POLD1 disrupts FBXW7α-mediated MYC ubiquitination and degradation

**Fig. 6:**
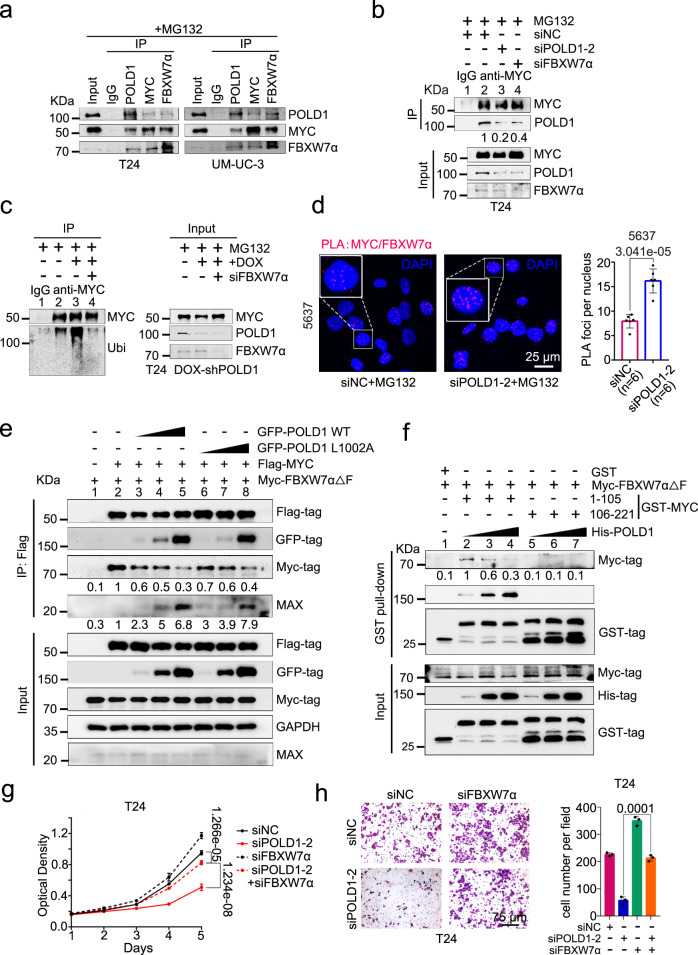
POLD1 supports MYC stability by interfering with FBXW7-mediated ubiquitination. **a** Western blot analysis of POLD1, MYC, and FBXW7α after IgG, POLD1-IP, MYC-IP, or FBXW7α-IP in T24 and UM-UC-3 cells. MYC was used as a positive control for FBXW7α endogenous interaction. All samples were treated with MG132 (10 μM) and incubated for 6 h before harvesting cells. **b** Western blot of POLD1 and MYC after IgG or MYC-IP in *POLD1* and/or *FBXW7α* knockdown T24 cells. All samples were incubated with MG132 (10 μM) for 6 h before harvest. **c** Western blot of ubiquitin and MYC after IgG or MYC-IP under denaturing conditions in DOX and/or *siFBXW7α* treatment in DOX-induced *shPOLD1* T24 cells. The cells of all samples were incubated with MG132 (10 μM) for 6 h before harvest. For the precipitates, the loading volume was adjusted to be equal to the precipitated MYC (**b**, **c**). **d** Representative images and statistics of PLA assays of MYC and FBXW7α in 5637 cells with or without *POLD1* depletion (*n* = 6 biologically independent experiments in each group). **e** Western blot of Flag-MYC, GFP-POLD1 and Myc-FBXW7α△F (F-box deletion mutant) after Flag-IP in 293T cells expressing GFP-POLD1 WT (0.1 μg, 1.0 μg, and 4.0 μg) or GFP-POLD1 L1002A (0.1 μg, 1.0 μg, and 4.0 μg) and Flag-MYC and Myc-FBXW7α△F. **f** The direct interaction between GST-MYC and His-POLD1 or Myc-FBXW7α△F was detected by GST pull-down assay. Recombinant GST-labeled fragments of MYC were incubated with lysates of 293T cells expressing Myc-FBXW7α△F and increased amounts of recombinant His-POLD1 (0.1, 1.0, and 3.0 μg). **g** The cell proliferation curve of T24 cells with *POLD1* and/or *FBXW7α* knockdown. *n* = 6 biologically independent experiments in each group. **h** Representative images and cell numbers of Transwell assays from the indicated groups with or without *POLD1* and/or *FBXW7α* knockdown in T24 cells (*n* = 3 biologically independent experiments in each group). Data are presented as the means ± SD (**d**, **g**, **h**). Statistical significance was determined by two-tailed Student’s *T*-test (**d**, **g**, **h**). IP-MS: Immunoprecipitation-mass spectrometry. PLA proximity ligation assay. Source data are provided as a Source Data file.

Our previous data revealed that POLD1 could bind to MYC and regulate its protein stability. Nevertheless, in terms of molecular function, the direct regulation of protein stability by POLD1 has not yet been reported. Our previous GO functional annotation of POLD1 showed that POLD1 was involved in the ubiquitin ligase binding process (Fig. 2f and Supplementary Table 2), so we hypothesized that POLD1 might interfere with the interaction between MYC and its key ubiquitin ligase, thereby affecting the protein stability of MYC. To validate this hypothesis, we screened E3 ubiquitin ligases that interact with POLD1 using the IP-MS assay (Supplementary Fig. 7a). The results showed that FBXW7 was the most likely E3 ubiquitin ligase to which POLD1 binds (Supplementary Dataset 2). In addition, MYC has been established to be predominantly regulated by FBXW7^32,33^ (by alternative splicing, FBXW7 can produce three protein isoforms: FBXW7α, FBXW7β, and FBXW7γ). FBXW7α was the most stable isoform with the highest expression level^46–48^, so FBXW7α was selected as a candidate molecule for subsequent assays. We confirmed interactions between POLD1 and FBXW7α using endogenous Co-IP assays in T24, 5637, and UM-UC-3 cells (Fig. 6a and Supplementary Fig. 7b). Moreover, we found that enhanced POLD1 strongly attenuated the effect of FBXW7α on the ubiquitination and half-life of MYC in 293T cells (Supplementary Fig. 7c, d). Notably, when *FBXW7α* was knocked down in T24 cells with *POLD1* depletion, we detected more POLD1 in immunoprecipitation than when *FBXW7α* was not knocked down (Fig. 6b, compare lane 4 vs lane 3). As expected, through endogenous ubiquitination assays, we found that *FBXW7α* knockdown in T24 cells counteracted the effect of DOX-induced *POLD1* knockdown on MYC ubiquitination (Fig. 6c, compare lane 4 vs lane 3). Furthermore, we found that *FBXW7α* knockdown in T24 cells with *POLD1* depletion notably rescued the loss of MYC abundance and stability induced by *POLD1* depletion (Supplementary Fig. 7e, f). Taken together, these results suggest that POLD1 stabilizes MYC by blocking the ubiquitination of MYC by FBXW7α, and this process is dependent on FBXW7α.

### POLD1 and FBXW7α competitively bind the MB1 domain of MYC

Since the previous results proved that POLD1 could directly bind to the MB1 domain of MYC (Fig. 5f, g), to which FBXW7α also binds, we speculated that POLD1 might competitively bind to the MB1 domain of MYC with FBXW7α. To prove this idea, we conducted a series of assays. We detected more PLA foci of MYC/FBXW7α in 5637 cells with *POLD1* depletion, which indicated that the colocalization of MYC and FBXW7α increased after *POLD1* depletion (Fig. 6d). Next, we examined the FBXW7α that MYC can bind to as the amount of POLD1 increases. We followed the strategy of Wang et al.^49^, using the FBXW7α F-box deletion mutant (FBXW7α△F) instead of FBXW7α WT, to exclude the interference of FBXW7α on the inhibition of MYC. After co-transfecting GFP-POLD1, Flag-MYC, and Myc-FBXW7α△F in 293T cells, we found that POLD1 was able to replace FBXW7α△F in the MYC-FBXW7α△F complex in a dose-dependent manner. Interestingly, with the increase in GFP-POLD1 transfection amount, the endogenous MAX that MYC can bind to increased significantly, indicating that POLD1 may promote the binding of MYC and MAX (Fig. 6e, compare lane 3–4 vs lane 2). As expected, we observed consistent results in the GST pull-down assays. With the increase in recombinant His-POLD1 protein amount, the binding FBXW7α△F of GST-MYC-1-105 (including the MB1 domain) decreased correspondingly. However, GST-MYC-106-221 (including the MB2 and MB3 domains) does not bind to POLD1 and FBXW7α△F (Fig. 6f, lanes 2–4). Of note, we used overexpressed GFP-POLD1 or GFP-FBXW7α 293T cell lysates to perform MST assays with MB1-T58A (theoretically not combined with FBXW7α) or MB1-T58D (it has a high affinity with FBXW7α) peptides, respectively. The results showed that the affinity of GFP-POLD1 for the MB1 (WT, T58A, T58D) peptide was greater than that of GFP-FBXW7α for it (Fig. 5e and Supplementary Fig. 7g). Finally, we found that the ability of POLD1 to affect T24 cell proliferation and metastasis was partially dependent on FBXW7α (Fig. 6g, h). Altogether, our results argued that POLD1 could stabilize MYC in a manner that competently binds with FBXW7α to the MB1 domain of MYC.

### POLD1 stabilizes MYC independent of DNA polymerase activity and p53

Considering that the DNA polymerase activity of POLD1 may have an effect on the stabilization of MYC, we constructed a POLD1 L1002A mutant confirmed to be deficient in DNA polymerase activity^8^ and conducted corresponding assays. We found that POLD1 L1002A has almost the same ability to stabilize MYC as POLD1 WT (Supplementary Fig. 7h). As expected, with the overexpression of POLD1 L1002A in 293T cells, the same amount of MYC in immunoprecipitation can bind less FBXW7α△F, and this phenomenon is almost consistent with POLD1 WT (Fig. 6e, compare lane 6–8 vs lane 3–5). Considering that *POLD1* knockdown theoretically causes DNA damage, we further investigated whether POLD1 stabilizes MYC through the activation of p53 signaling. The depletion of *p53* in T24 cells with *POLD1* depletion had a minimal effect on MYC abundance compared to the absence of *p53* depletion (Supplementary Fig. 7e). Furthermore, we found that *p53* knockdown only minimally rescued the proliferation and metastasis ability of T24 cells lost due to *POLD1* depletion (Supplementary Fig. 8a, b). In summary, POLD1 stabilizes MYC in a manner independent of its enzyme activity and p53 signaling.

### POLD1 stabilizes MYC depending on the C-terminal domain of POLD1 and T58 of MYC

The previous results demonstrated that MYC can bind to both the N-terminal and C-terminal of POLD1. Further assays were conducted to understand which terminal was responsible for stabilizing MYC. We overexpressed full-length, N-terminal and C-terminal POLD1 in 293T cells and showed that the C-terminal of POLD1, but not the N-terminal, increased MYC abundance and stability and reduced FBXW7α-induced MYC ubiquitination (Supplementary Fig. 8c–e). It is consistent that the effects of POLD1 on proliferation and metastasis were also mainly dependent on the C-terminal of POLD1 (Supplementary Fig. 8f, g). We subsequently explored whether POLD1 could affect the regulatory mechanisms upstream of FBXW7α. Phosphorylation of S62 and T58 is well recognized to occur before FBXW7α-induced ubiquitination of MYC^50,51^. Western blot analysis of pS62 and pT58 was performed in T24 cells with *POLD1* knockdown. The results showed that pS62 was significantly downregulated compared with total MYC, while pT58 was increased (Supplementary Fig. 8h). Similar to previous results, *POLD1* depletion reduced total MYC protein abundance. However, MYC-T58A, which cannot be recognized by FBXW7α^52^, significantly rescued the effect of *POLD1* knockdown on MYC and the phenotype (Supplementary Fig. 8i, j). Altogether, this result suggests that POLD1 regulation of MYC stability depends on the C-terminal domain of POLD1 and phosphorylation of T58 of MYC.

### POLD1 forms a complex with MYC to affect the transcriptional activity of MYC

**Fig. 7:**
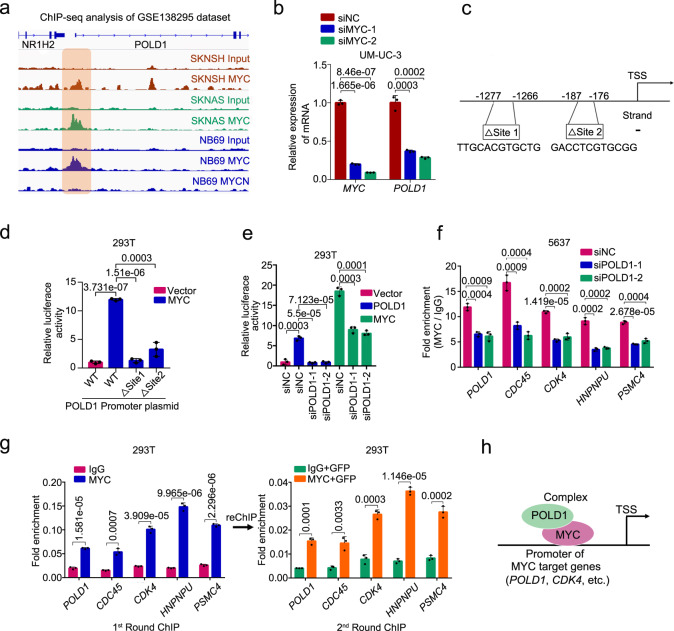
POLD1 affects the transcriptional activity of MYC and is the target gene for transcriptional activation of MYC. **a** Genome browser tracks of MYC or MYCN occupancy in the *POLD1* loci in SKNSH, SKNAS or NB69 cells (public dataset: GSE138295^53^). The genome browser map is displayed by IVG software. The brown region marks a region in the *POLD1* promoter region where MYC is significantly enriched relative to input. **b** Effects of *MYC* depletion on *POLD1* transcription levels in UM-UC-3 cells. **c** Schematic diagram of the two MYC binding sites in the *POLD1* promoter region predicted by the JASPAR website. **d** Luciferase reporter assays of the *POLD1* promoter in Vector, Flag-MYC, and WT *POLD1* promoter or mutant *POLD1* promoter (△Site 1 and △Site 2)-overexpressing 293T cells. **e** Luciferase reporter activities of the *POLD1* promoter were assessed in the presence of exogenous MYC or POLD1 in *POLD1*-depleted 293T cells. **f** ChIP-qPCR assays of MYC binding to the *POLD1*, *CDC45*, *CDK4*, *HNRNPU* and *PSMC4* promoters in 5637 cells with or without *POLD1* depletion. *CDC45*, *CDK4*, *HNRNPU* and *PSMC4* were both confirmed as MYC target genes and were shown to be significantly downregulated in 5637 cells with *POLD1* knockdown in previous results. **g** ChIP-reChIP assays: The first round of ChIP analysis of binding of IgG or MYC to the promoter of target genes of MYC in 293T cells overexpressing GFP-POLD1, the second round of ChIP analysis of binding of GFP-POLD1 to the promoter target genes of MYC in the eluent for the first round of ChIP assay. **h** Schematic illustration of POLD1 and MYC binding as complexes to promoters of target genes of MYC to promote transcription. Statistical significance was determined by two-tailed Student’s *T*-test (**b**, **d**–**g**). Data shows mean ± SD, *n* = 3 biologically independent experiments in each group (**b**, **d**–**g**). TSS Transcription start site. Source data are provided as a Source Data file.

MYC is a well-established transcription factor key in activating the transcription of most genes. We analyzed the ChIP-seq data in the public database (GSE138295^53^) and found that MYC, but not MYCN, has a significant signal in the promoter region of *POLD1* (Fig. 7a). RNA-seq data from public databases (GSE126739^35^) showed that *POLD1* transcription levels were significantly reduced after *MYC* knockdown (Supplementary Fig. 9a). Next, RT-qPCR and immunoblot assays exhibited that the transcription and protein expression of POLD1 were notably reduced after *MYC* depletion (Fig. 7b and Supplementary Fig. 9b). Subsequently, two *POLD1* promoter mutations (△site1 and △site2) were constructed according to the MYC binding motif predicted by the JASPAR database (Fig. 7c and Supplementary Fig. 9c). The results of the luciferase assays substantiated that MYC significantly activated the transcription of *POLD1* compared with the vector control or two *POLD1* promoter mutants (Fig. 7d). Furthermore, luciferase assays showed that *POLD1* knockdown in 293T cells significantly inhibited MYC activation of POLD1 promoter activity (Fig. 7e). Next, we detected in 5637 cells by ChIP-qPCR assays that after *POLD1* depletion, the promoter DNA fragments of MYC target genes (*POLD1*, *CDC45*, *CDK4*, *PNRNPU*, and *PSMC4*) that MYC could bind to were sharply reduced (Fig. 7f). These results indicate that *POLD1* depletion can significantly reduce MYC transcriptional activity.

Interestingly, the luciferase assays showed that POLD1 also enhanced its promoter activity, while *POLD1* knockdown counteracted this effect (Fig. 7e). Since it is still not clear whether POLD1 itself can regulate gene transcription, combined with the previous results, we speculated that POLD1 might form a complex with MYC to participate in transcriptional regulation. To confirm this hypothesis, we further conducted reChIP assays. Since there was no ChIP-grade POLD1 antibody available, we overexpressed GFP-POLD1 in 293T cells and then conducted follow-up assays with ChIP-grade GFP antibody. As expected, after the first round of ChIP, we detected more enrichment of MYC target gene promoters, including *POLD1*, in MYC-ChIP than in IgG. Moreover, after the second round of GFP-ChIP, we found that GFP-POLD1 can also bind to the promoter of MYC target genes, including itself (Fig. 7g and Supplementary Fig. 9d). Combining the above results, we believe that POLD1 can bind MYC in the form of a complex at promoters of MYC target genes including *POLD1* and increase the transcriptional capacity of MYC (Fig. 7h).

Similarly, *MYC* depletion can also cause proliferation and cell cycle disturbances. We constructed a DOX-induced *MYC* knockdown stable cells using the same strategy as before. The results showed that *MYC* was notably inhibited 12 h after DOX administration, while the target genes of MYC, including *POLD1*, were significantly downregulated at 24 h (Supplementary Fig. 9e). The protein abundance of both MYC and POLD1 was depleted 24 h after DOX administration (Supplementary Fig. 9f, g), while the cell cycle was arrested in G1 phase (Supplementary Fig. 9h). Then proliferation ability was impaired 36 h after DOX administration (Supplementary Fig. 9i). Based on the above results, the cell cycle changes caused by *MYC* knockdown occurred at the same time as the consumption of POLD1 protein level (Supplementary Fig. 9j), which cannot rule out the possibility that the cell cycle disturbance caused by *MYC* knockdown may affect POLD1.

**Fig. 8:**
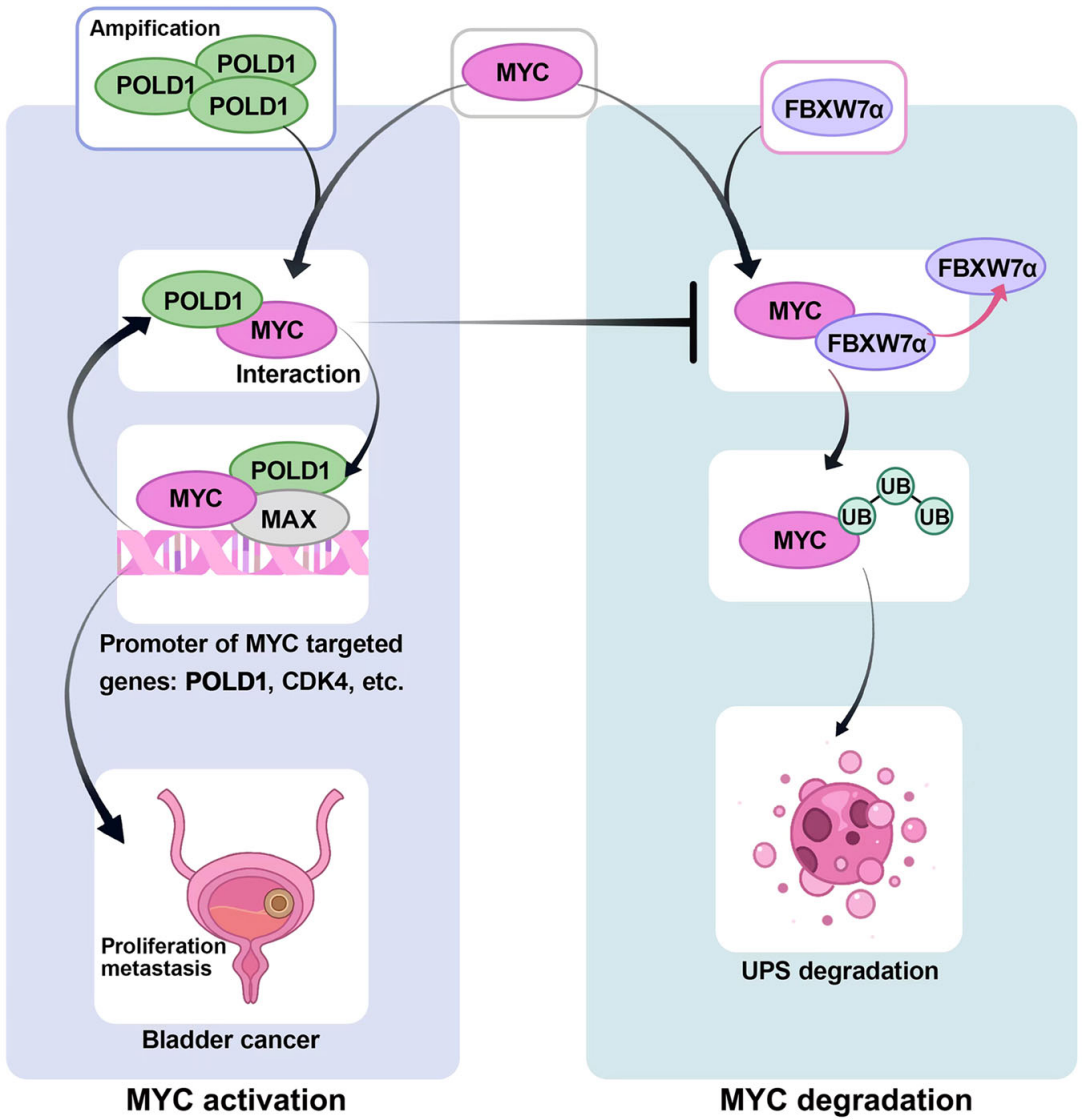
Mechanism diagram of this study. The schematic diagram illustrates the POLD1-MYC axis driving the proliferation and metastasis of BLCA. In BLCA, *POLD1* is upregulated due to amplification, *MYC* transcriptional activation and other causes. POLD1 competitively binds MYC with FBXW7 to reduce the ubiquitination degradation of MYC. Thus, POLD1 upregulates MYC and promotes the proliferation and metastasis of BLCA. At the transcriptional level, POLD1 can bind with MYC in the form of a complex to the promoter of the MYC target gene to increase the transcriptional capacity of MYC. In addition, MYC can also bind to the promoter region of *POLD1* and activate *POLD1* transcription. Thus, a POLD1-MYC positive feedback loop is formed to promote the proliferation and metastasis of BLCA. Ub ubiquitin, UPS ubiquitin-proteasome system.

In conclusion, we identified a mechanism by which POLD1 regulates the proliferation and metastasis of BLCA mediated by the stabilization of MYC. Specifically, as shown in Fig. 8, *POLD1* was upregulated due to amplification or MYC transcriptional activation and competed with FBXW7α to bind MYC, leading to weakened MYC ubiquitination, preventing FBXW7α from binding MYC, and finally stabilizing MYC. On the other hand, *POLD1* is the transcriptional target of MYC and can form a complex with MYC to participate in the transcriptional regulation of MYC, thus promoting the transcriptional activity of MYC. Finally, a POLD1-MYC positive feedback loop is formed, which accelerates the deterioration of BLCA.

## Discussion

In earlier research, we found that POLD1 may play a role in BLCA tumorigenesis^6^. This study elucidated the effect of POLD1 on BLCA from the carcinogenic mechanism of abnormally high expression of *POLD1*. Evidence suggests that *POLD1* is upregulated in most tumors, partly due to *POLD1* amplification. However, it is unclear whether *POLD1* amplification has a carcinogenic effect^54^. In most tumors, the main factors leading to *POLD1* upregulation and whether *POLD1* upregulation is carcinogenic have not been clarified. Herein, we expounded on the pro-carcinogenic role of POLD1 in BLCA. We found that POLD1 was significantly associated with the pathological grade of BLCA in both NMIBC and MIBC cohorts. The higher the pathological stage and T stage, the higher the protein level of POLD1, and POLD1 was substantially higher in MIBC than in NMIBC. Although POLD1 is not a particularly excellent prognostic marker, it still has some reference value. Furthermore, we provided compelling evidence that POLD1 promotes BLCA proliferation and metastasis by stabilizing MYC in a DNA-independent manner. We proposed a previously unreported oncogenic mechanism of POLD1, and we found that among the four subunits of DNA polymerase (*POLD1*, *POLD2*, *POLD3*, and *POLD4*), only *POLD1* was significantly upregulated in BLCA, indicating that *POLD1*’s effect on BLCA may be independent of its enzyme activity and has some specificity when compared to other subunits. Notably, *POLD1* is activated by MYC at the transcriptional level, forming a positive feedback loop with MYC. This mutual activation between POLD1 and MYC is critical for maintaining mutual overexpression, thereby enhancing the pro-carcinogenic effect of POLD1-MYC on BLCA.

Ample evidence substantiates that MYC is abnormally expressed in almost all tumors, especially hematological tumors, and amplification is one of the reasons for abnormally high *MYC* levels in BLCA^55–58^. However, studies have reported a low IHC positive rate of MYC in high-grade BLCA^59^, which may be caused by the high level of MYC-inducing cell apoptosis^60^. The protein level of MYC is strictly regulated by E3 ubiquitin ligase. FBXW7 has been reported as one of the most important E3 ligase for MYC degradation and is dependent on the T58 phosphorylation of MYC^34,61^. However, the mutation of T58 to a non-phosphorylated residue stabilized MYC more than it depleted *FBXW7*^52^, indicating that additional components are involved in the FBXW7-mediated ubiquitination of MYC. The present study identified a mechanism that controls MYC degradation whereby POLD1 stabilizes the MYC protein by blocking MYC ubiquitination by FBXW7 through its non-enzymatic function. This process of POLD1 stabilizing MYC is substantially dependent on the C-terminal domain of POLD1, and through in vivo and in vitro assays, we demonstrated that POLD1 and FBXW7 mainly compete with the MB1 domain of MYC. This also complements the cause of the upregulation of *MYC* in addition to *MYC* amplification. In our study, the depletion of *POLD1* significantly reduced the expression of Cyclin D1 and Cyclin E1, and rescue assays validated the importance of MYC for this process. Furthermore, Cyclin D1 and Cyclin E1 can promote the cell cycle transition from G1 to S phase^42–44^. In conclusion, POLD1 can accelerate the cell cycle progression through the MYC/Cyclin D1/Cyclin E1 axis, promoting the proliferation of BLCA cells. However, even though our experiments showed that the influence of POLD1 on the stability of MYC occurred before the influence of POLD1 on phenotypic changes, we could not completely rule out the influence of phenotypic changes on MYC.

EMT is considered a key driver of cancer cell spread and invasion, involving transcription factors in the Snail, Twist, and Zeb families^62,63^. In our study, *POLD1* downregulation significantly reduced the expression of Snail and its downstream EMT-related protein, and *MYC* overexpression countered this process to some extent. Indeed, *SNAIL* is the target gene of MYC transcriptional regulation^64,65^. There are some deficiencies in our studies related to in vivo metastasis; that is, the tail vein injection lung metastasis assay could not exclude the effect of proliferation differences. We will further elucidate the effect of POLD1 on BLCA metastasis in vivo in future studies. Taken together, POLD1 regulates the EMT process of BLCA through the MYC/Snail axis to promote the metastatic ability of BLCA.

In conclusion, we propose a POLD1-MYC regulatory mechanism for MYC-driven BLCA, and POLD1 has potential as a biomarker for BLCA.

## Methods

### Ethical statement

This study was performed in accordance with the Declaration of Helsinki and was approved by the Institutional Ethics Committee of Zhongnan Hospital of Wuhan University (approval number: 2020102) and Experimental Animal Welfare and Ethics of Zhongnan Hospital (approval number: ZN2021005). In our study, no tumor size exceeded the 2 cm limit required by the Ethics Committee. Bioinformatics data involving humans were collected from publicly available databases with anonymous patient information.

### Human BLCA tissues

The human BLCA tissues (MIBC) and paired paracancerous tissues (*n* = 12 per group) used in this study were obtained from the Department of Urology, Zhongnan Hospital of Wuhan University, Wuhan, China. The study was approved by the Institutional Ethics Review Board (approval number: 2020102). The tissue chips (HBlaU079Su01, *n* = 79) used in this study were purchased from Shanghai Outdo Biotech Co., Ltd. (http://www.superchip.com.cn/index.html). Written informed consent of all individuals has been obtained.

### Cell culture and transfections

SV-HUC-1, RT4, UM-UC-3, T24, 5637, J82, SCaBER and HEK293T cells were kindly provided by the Cell Bank of the Chinese Academy of Science (Shanghai, China). T24, 5637, SV-HUC-1, RT4, ScaBER and J82 cells were cultured and maintained in RPMI 1640 medium supplemented with 10% fetal bovine serum. UM-UC-3 was cultured and maintained in MEM supplemented with 10% fetal bovine serum. HEK 293T cells were cultured and maintained in DMEM medium supplemented with 10% fetal bovine serum. Authentication was performed by Cell Bank, Chinese Academy of Sciences (Shanghai, China). All cell lines were tested negative for mycoplasma contamination. Transfection of siRNA and plasmid followed Lipofectamine^TM^ 3000 Reagent’s (L3000015, Invitrogen) protocol.

### Antibodies

Detailed information on the antibodies used in this study is listed in Supplementary Table 3.

### siRNAs and plasmid construction

All siRNAs (*siPOLD1-1*: 5′-GUUGGAGAUUGACCAUUAUTT-3′, *siPOLD1-2*: 5′- GGUGGAGUCUAAGUACACATT-3′, *siPOLD2-1*: 5′-GUUGGAGAUUGACCAUUAUTT-3′, *siMYC-1*: 5′-GCUUGUACCUGCAGGAUCUTT-3′, *siMYC-2*: 5′-GGAAGAAAUCGAUGUUGUUTT, *siFBXW7α*: 5′-GCATATGATTTTATGGTAA-3′, *sip53*: 5′-AAUAUUCUCCAUCCAGUGGTT-3′) used in this study were purchased from GenePharma (Shanghai, China). The human MYC-HA-tagged, MYC-N-terminal-HA-tagged (amino acids 1-220), MYC-C-terminal-HA-tagged (amino acids 221-439), MYC-T58A-HA-tagged, MYC-Flag-SBP-tagged, MYC△MB1 (deletion MYC MBI mutant)-Flag-SBP-tagged, MYC△MB2 (deletion MYC MBII mutant)-Flag-SBP-tagged, MYC△MB3 (deletion MYC MBIII mutant)-Flag-SBP-tagged, FBXW7α△F (F-box deletion mutant)-Myc-tagged and FBXW7α-Flag-tagged plasmids were kindly provided by Prof. Guoliang Qing and Prof. Hudan Liu at Wuhan University, China. Human POLD1-GFP-tagged plasmids were purchased from GeneCopoeia (Guangzhou, China). POLD1-N-terminal-GFP-tagged (amino acids 1-984), POLD1-C-terminal-GFP-tagged (amino acids 985-1107) and POLD1-Flag-tagged were constructed by standard subcloning. pGL4.10-POLD1 promoter-WT (POLD1 WT), pGL4.10-POLD1 promoter-deletion mutation 1 (△Site 1: TTGCACGTGCTG), pGL4.10-POLD1 promoter-deletion mutation 2 (△Site 2: GACCTCGTGCGG) and pGL4.10-Renilla were purchased from OBiO Technology (Shanghai, China) Co., Ltd.

### Protein purification

Recombinant GST, His-POLD1, and GST-MYC proteins were purchased from AtaGenix (Wuhan, China). The experimental procedures are briefly described as follows: full-length and fragment POLD1 and MYC were subcloned into vectors containing His-tag or GST-tag, respectively. They were expressed in *E. coli* cells or insect cells and purified by affinity chromatography. The fractions on the column were collected, concentrated, and purified by gel filtration chromatography.

### Immunoprecipitation-mass spectrometry (IP-MS) analysis

Flag-Vector and Flag-POLD1 plasmids were transfected into 293T cells for 24 h, followed by immunoprecipitation. The reaction solution containing sodium deoxycholate (SDC), tris (2-carboxyethyl) phosphine (TCEP), and chloroacetamide (CAA) was added to the magnetic beads for one-step reduction, alkylation, and elution. This step was repeated twice, combined with the eluent, diluted with water, and then added into trypsin for enzymatic hydrolysis overnight. After enzymatic hydrolysis, the peptide solution is desalted through the desalting column. After being drained by centrifuge concentrator, the sample was frozen at −80 °C for mass spectrometer testing. Mass spectrometry was performed using an Orbitrap Exploris 480 liquid chromatography-mass spectrometry (LC-MS) system (Thermo Fisher Scientific, USA). The results generated by LC-MS were retrieved by MaxQuant (V1.6.2.10), and the database retrieval algorithm was MaxLFQ.

### MTT assays

The transfected cells were planted in 96-well plates at 3000 cells per well for 24 h at 37 °C, and MTT reagent (methyl thiazolyl tetrazolium, Sigma) was added to the cell culture medium at 20 µL per well for 4 h at 37 °C. Then the medium was discarded, and 200 µL DMSO was added to each well and placed on a shaker for 10 min. Finally, the absorbance of the sample was measured with a microplate reader. Perform the test in the above manner for five consecutive days.

### Flow cytometry

Transfected BLCA cells were harvested and washed twice with cold PBS, followed by centrifugation. The cells were then suspended using propidium iodide (PI, 100 μg/mL) and permeabilization solution in the cell cycle staining kit (CCS012, Multi sciences) and incubated in the dark for 30 min at room temperature. Finally, flow cytometry (Beckman Cytoflex) was used to detect the samples and FlowJo software (version 7.6) was used to analyze the results. The FACS sequential gating strategies are shown in Supplementary Fig. 10.

### Colony formation assays

The transfected cells were planted at 1000 cells per well in 6-well plates and placed in a cell culture box at 37 °C for approximately one week. The culture medium was discarded, and the cells were fixed with 4% formaldehyde for 30 min, stained with 0.1% crystal violet for 30 min, washed with clean water and dried.

### Migration and wound healing assays

For transwell assays, the cells were placed in serum-free medium, and then a certain number of cells (T24 cells: 3 × 10^4^ cells per well, 5637 cells: 1.2 × 10^5^ cells per well) were added to the transwell chamber for migration assays. For the wound healing assay, when the density of cells in the 6-well plates reached nearly 100%, the cells were scratched and photographed. After 24 h, photographs of the scratched area were taken again. We measured the distance between the leading edges of the cells after the scratch using Photoshop CC (2018 version). Gap closure (%) = (0 h distance−24 h distance)/0 h distance × 100%.

### Immunofluorescence

One day before the assay, the cells were placed in a 6-well plate covered with 22 × 22 mm cover glass (sterilized beforehand). The cell density was adjusted to approximately 30%, and the colocalization assay was even lower. Cells were immobilized with fresh 4% paraformaldehyde for 20 min, washed with PBS three times, and then treated with 0.4% Triton X-100 for 10 min. The cells were washed three times with PBS again. The sections were sealed with 2% BSA for 30 min and washed twice with PBS. The configured primary antibody was added and incubated overnight at 4 °C. The secondary antibody was added and incubated at room temperature for 1 h, and then 0.5 μg/ml DAPI (prepared in PBS) was added for staining for 10 min. Images were captured using a confocal laser microscope (C2^+^, Nikon, Japan). Details of the antibodies used in this experiment are listed in Supplementary Table 3.

### Immunohistochemistry

For the collected tissue samples, formalin fixation, paraffin embedding, section dewaxing, hydration, serum blocking, and incubation with primary and secondary antibodies were performed in turn. Finally, fresh DAB color solution was added and placed under a microscope for observation. Details of the antibodies used in this experiment are listed in Supplementary Table 3.

### RNA isolation and quantitative real-time PCR analyses

We used a HiPure Total RNA Mini Kit (R4111-03, Magen, China) to isolate RNA from cells. We used a NanoPhotometer (Cat. #N60, Implen, Germany) to detect the quality and concentration of the extracted RNA. We obtained cDNA according to the protocol for the ReverTra Ace qPCR RT Kit (FSQ-101, TOYOBO, Japan). Real-time quantitative PCR (qRT-PCR) was performed by the iQ™ SYBR® Green Supermix system (Bio-Rad, China). The primers used for qPCR in this study are listed in Supplementary Table 4.

### Immunoblot and immunoprecipitation analysis

For immunoblotting, cells were collected and lysed using a 50:1:1 mixture of RIPA buffer, phenylmethanesulfonyl fluoride (PMSF, 1 mM) and Roche phosphatase inhibitors on ice. Protein content was measured using a BCA assay kit (Thermo Fisher Scientific). Whole cell lysates were separated by SDS-PAGE, and then the protein was transferred to a polyvinylidene difluoride (PVDF) membrane. After being blocked with 5% skim milk in TBST buffer (10 mM Tris-HCI, 150 mM NaCI, and 2% Tween 20), the corresponding primary antibody was added and incubated overnight at 4 °C. The next day, the PVDF membrane was washed three times with TBST buffer. Then, 2% skim milk was incubated with a secondary antibody solution at room temperature for 1 h. TBST was used again to wash the PVDF membrane three times. Finally, the proteins were detected by chemiluminescence and gel imager (ChemiDoc XRS, Bio-Rad, USA).

The Co-IP assay was performed using BeaverBeads^TM^ Protein A (or A/G) Immunoprecipitation Kit (22202-100, Beaver, China). The short protocol is described below: Cells in 6-well plates were collected (exogenous IP: 1 × 10^6^ 293 T cells; endogenous IP: 1 × 10^7^ T24, 5637 or UM-UC-3 cells), and IP binding buffer (500 ml PBS, 0.3% Tween 20, and 75 mM NaCl) was added for lysis according to the amount of 1 mL in each well on ice for 40 min. The supernatant obtained after centrifugation was used as the samples of input, IgG and IP respectively. The recommended dose of target antibody was added and incubated overnight at 4 °C. The next day, the magnetic beads were washed three times with IP binding buffer, and each sample was incubated with 20 μL of magnetic beads at 4 °C for 2 h. After that, the supernatant was removed and the magnetic beads were washed again three times. Then, 100 μL loading buffer was added, mixed with magnetic beads, and heated at 100 °C for 6 min to denature. Finally, a Western blot assay was used to detect the protein content. For the GST pull-down assay, the purified proteins (2 μg) were added to IP binding buffer according to the specified grouping. Each sample was added to 20 μL glutathione-Sepharose magnetic beads and incubated overnight at 4 °C. The beads were washed several times using IP binding buffer, followed by denaturation of the proteins at 100 °C for 6 min. Immunoblotting was used to measure the protein level. Details of the antibodies used in this experiment are listed in Supplementary Table 3.

### Protein half-life assays

Cycloheximide (CHX) at a final concentration of 50 μg/mL was added to the cell culture medium at 37 °C, the cells were collected at the specified time points, proteins were extracted, and finally, the target proteins were detected by immunoblot. The target protein was quantitatively analyzed by ImageJ software (version 1.52), and the results were displayed by GraphPad Prism software (version 7). Proteins quantified in all CHX assays were normalized to the loading control.

### Luciferase reporter assays

293T cells were planted on 12-well plates. When the cell density was approximately 60%, the plasmid with the specified combination was transfected into 293T cells for 24 h. Renilla luciferase was used as a control. Specific experimental details followed the protocol of the Dual-luciferase Reporter Assay System kit (Promega).

### Chromatin immunoprecipitation (ChIP) and reChIP assay

We used the SimpleChIP Plus Sonication Chromatin IP Kit (Cell Signaling Technology, #56383) to perform ChIP-related assays. Briefly, 1 × 10^7^ treated cells were collected, cross-linked with 1% paraformaldehyde for 10 min at room temperature, and then quenched with 0.125 M glycine for 5 min. Cells were lysed with ChIP Sonication Cell Lysis Buffer for 30 min at 4 °C on a vertical rotary shaker. The lysed cells were broken by ultrasound using ChIP Sonication Nuclear Lysis Buffer, so the DNA fragment size was 300–500 bp. The chromatin fragments were immunoprecipitated with MYC or IgG antibody at 4 °C overnight. The chromatin-antibody complex was then incubated with magnetic beads for 3 h at 4 °C. Chromatin was eluted from the beads using the elution buffer, and the results were quantified using a qPCR instrument.

For the reChIP assay, we performed a first round of ChIP using MYC or IgG antibodies. The eluent obtained from the first round of ChIP was incubated with GFP antibody at 4 °C overnight. The subsequent method was the same as above. All primer information related to the ChIP and reChIP assays is listed in Supplementary Table 4. Details of the antibodies used in this experiment are listed in Supplementary Table 3.

### Proximity ligation assay (PLA)

The previous procedure was consistent with immunofluorescence, and after incubating overnight with the corresponding primary antibody, we washed the cells three times with wash buffer (Sigma, DUO82049). The cells were then incubated with the secondary antibody with the PLA probe (Sigma, anti-mouse PLUS probe: DUO92001-30RXN; anti-rabbit MINUS probe: DUO92005-30RXN) at room temperature for 1 h. The cells were washed three times with PBS again. The cells were then incubated with the Duolink® In Situ Red detection reagent (Sigma, DUO92008, Germany) at room temperature for 2 h. Finally, the cells were stained with 0.5 μg/mL DAPI (prepared in PBS) and covered onto slides. Images were captured by confocal laser microscopy.

### Microscale thermophoresis (MST) assay

MST is a biomolecular interaction analysis technology. It measures the affinity between a ligand and a target molecule by observing the changes in its conformational size, charge, and solvation state after binding to the target molecule^66^. We overexpressed GFP-POLD1 or GFP-FBXW7α in 293T cells and then lysed the collected cells with IP lysis buffer. The affinity between the whole cell lysate and the MBI peptides (WT: APSEDIWKKFELLP**T**PPLSP, T58A: APSEDIWKKFELLP**A**PPLSP, T58D: APSEDIWKKFELLP**D**PPLSP) was detected by a Monolith NT.115 instrument (NanoTemper). The concentration of MBI peptide was set as follows: 2.5 mM, 0.5^1^ × 2.5 mM, 0.5^2^ × 2.5 mM, 0.5^3^ × 2.5 mM, 0.5^4^ × 2.5 mM, 0.5^5^ × 2.5 mM, 0.5^6^ × 2.5 mM, 0.5^7^ × 2.5 mM, 0.5^8^ × 2.5 mM, 0.5^9^ × 2.5 mM, 0.5^10^ × 2.5 mM, 0.5^11^ × 2.5 mM, 0.5^12^ × 2.5 mM, 0.5^13^ × 2.5 mM, 0.5^14^ × 2.5 mM and 0.5^15^ × 2.5 mM. The data were obtained from three independent repeated experiments, and the results were displayed by GraphPad Prism 7.0.

### Cell synchronization analyses

T24 cells at 25% confluency were planted in a tissue culture dish, and thymidine (2 mM) was added for 18 h. The thymidine was removed by washing three times with PBS, and fresh medium was added for 9 h. Thymidine was added for 15 h. The cells were released by washing three times with PBS, and fresh medium was added. From this point, cells were collected at intervals for subsequent analysis.

### Animal experiments

T24, 5637 or UM-UC-3 cells were transfected with lentivirus purchased from GenePharma (Shanghai), and stable *POLD1* knockdown cells (*shPOLD1*) or negative control (*shNC*) cells were screened by puromycin (Sigma, 1 μg/mL). The mice used in the study were all male because the incidence of BLCA is much higher in men than in women^67^. Four-week-old male nude mice (NOD/SCID) purchased from Beijing HFK Bioscience Co., Ltd. were adaptively fed for one week. For the subcutaneous tumor-bearing assay, 1 × 10^7^ *shNC* and *shPOLD1* cells were subcutaneously injected into nude mice (NOD/SCID). After almost two weeks, the size of the tumor was measured every three days. The formula for estimating tumor volume is V = 1/2 × L × S^2^ (L: long diameter, S: short diameter). For the tail vein injection lung metastasis assay, we collected 1 × 10^6^ *shNC* and *shPOLD1* cells, resuspended them in 100 μL of PBS, and injected them into the tail of 4-week-old nude mice. Fluorescence detection was performed after normal feeding for approximately 8 weeks. The nude mice were sacrificed, and the tumor tissue was separated, weighed, and photographed, followed by immunohistochemistry and H&E staining of the tumor tissue. All work with mice was approved by and performed under the regulations of the Experimental Animal Welfare and Ethics Committee of Zhongnan Hospital (approval number: ZN2021005).

### RNA-seq and bioinformatics processing

For the RNA-seq assay, we transfected *siPOLD1* into 5637 cells for two days, collected cells, extracted RNA, and conducted RNA quality control with a NanoDrop ND-1000 instrument. A library with a fragment size of 300 bp ± 50 bp was obtained by constructing the library. Finally, we performed paired-end sequencing on an Illumina Novaseq™6000 (LC-Bio Technology) according to the vendor’s recommended protocol. HISAT2 software was used to map reads to the genome (Homo sapiens Ensembl v96). StringTie software was used for the initial assembly of a gene or transcript. The Gffcompare software was used to obtain the final assembly comment result. Finally, the “ballgown” package was used to provide the file input for transcripts per kilobase million (TPM) quantification.

The differentially expressed genes of RNA-seq were analyzed by R package “DEseq2”, and the screening threshold was adjusted *p* < 0.05. We used Pearson correlation analysis in the TCGA-BLCA dataset to screen for protein-coding genes that were significantly positively associated with *POLD1*. The screening threshold was set to *p* < 0.05 and the Pearson correlation coefficient > 0. The R package “clusterProfiler” was used to perform gene set enrichment analysis (GSEA), Gene Ontology (GO). GSEA was performed on 50 cancer hallmark gene sets and was conducted by ranking the Pearson correlation coefficients with *POLD1* expression from highest to lowest. *p* < 0.05 was considered statistically significant.

### Statistics and reproducibility

In this study, the Shapiro-Wilk normality test^68^ was used to test the normality of data. For the comparison of the two groups of data, a paired/unpaired *T*-test was used for analysis if the data presented a normal distribution, and the Wilcoxon rank-sum test was used for analysis if the data did not conform to a normal distribution. For the comparison of data above the two, if the data conformed to the normal distribution, analysis of variance was used for the test. Otherwise, Kruskal–Wallis tests were used. Survival analysis was statistically analyzed by the log-rank test. The experiments were independently repeated three times with similar results (Figs. 5a, b, h, 6a–c, e–f, Supplementary Fig. 6, Fig. 7d, Fig. 9b, Fig. 9d). All statistical analyses were performed by R (version 4.1.2) software.

### Reporting summary

Further information on research design is available in the Nature Portfolio Reporting Summary linked to this article.

## Supplementary information

Supplementary Information

Reporting Summary

Supplementary Dataset 1

Supplementary Dataset 2

Description of Additional Supplementary Files

## Data Availability

The mass spectrometry proteomics data generated in this study have been deposited in the ProteomeXchange database under accession code PXD040806, the results of mass spectrometry assays generated in this study are provided in Supplementary Dataset 2. The RNA-seq data generated in this study have been deposited in the GEO database under accession code GSE200897. The publicly available GSE13507^38^ cohort data (The data included 165 samples of primary BLCA, 23 recurrent NMIBC, 58 paracancerous tissues, and 10 normal tissues. Among them, 165 cases of primary BLCA with matching clinical data were selected as the study object) used in this study are available in the GEO database under accession code GSE13507, The publicly available GSE32894^40^ cohort data (RNA-seq data from 224 urothelial tissue samples in this cohort and matching clinical follow-up data were selected for our analysis) used in this study are available in the GEO database under accession code GSE32894. The publicly available GSE126739^35^ cohort data (these RNA-seq data contained three replicates of *siNC* and *siMYC* processing in the Jurkat cell line) used in this study are available in the GEO database under accession code GSE126739, The publicly available GSE138295^53^ cohort data (these data include the MYC and MYCN ChIP-seq data for four neuroblastoma cell lines) used in this study are available in the GEO database under accession code GSE138295. The publicly available TCGA-BLCA cohort data (the data included 393 MIBC, 6 NMIBC, and 19 normal samples) were obtained from the UCSC Xena website (https://xenabrowser.net/). The publicly available UROMOL^39^ cohort data (the data included 16 MIBC, 460 NMIBC and matching clinical follow-up data) were downloaded from ArrayExpress website (https://www.ebi.ac.uk/biostudies/arrayexpress/studies/E-MTAB-4321?query=UROMOL%20#). The details of the cohorts used in our study are listed in Supplementary Tables 5–9. The mutation data of Supplementary Fig. 1a used in our study were downloaded from the cBioPortal website (https://www.cbioportal.org/). The remaining data are available within the Article, Supplementary Information or Source Data file. Source data are provided as a Source Data file. Source data are provided with this paper.

## Source data

Source Data
