# Supplementary material for: DNA polymerase POLD1 promotes proliferation and metastasis of bladder cancer by stabilizing MYC
Source: Nat Commun. 2023 Apr 27;14:2421. doi: 10.1038/s41467-023-38160-x (PMC10140023; doi:10.1038/s41467-023-38160-x)
Supplement: Supplementary file 1 — Supplementary Information [file 41467_2023_38160_MOESM1_ESM.pdf]

Supplementary Information

DNA polymerase POLD1 promotes proliferation and metastasis of bladder cancer by stabilizing MYC

Yejinpeng Wang, Lingao Ju, Gang Wang, Kaiyu Qian, Wan Jin, Mingxing Li, Jingtian Yu, Yiliang Shi, Yongzhi Wang, Yi Zhang, Yu Xiao, Xinghuan Wang

Supplementary Figures

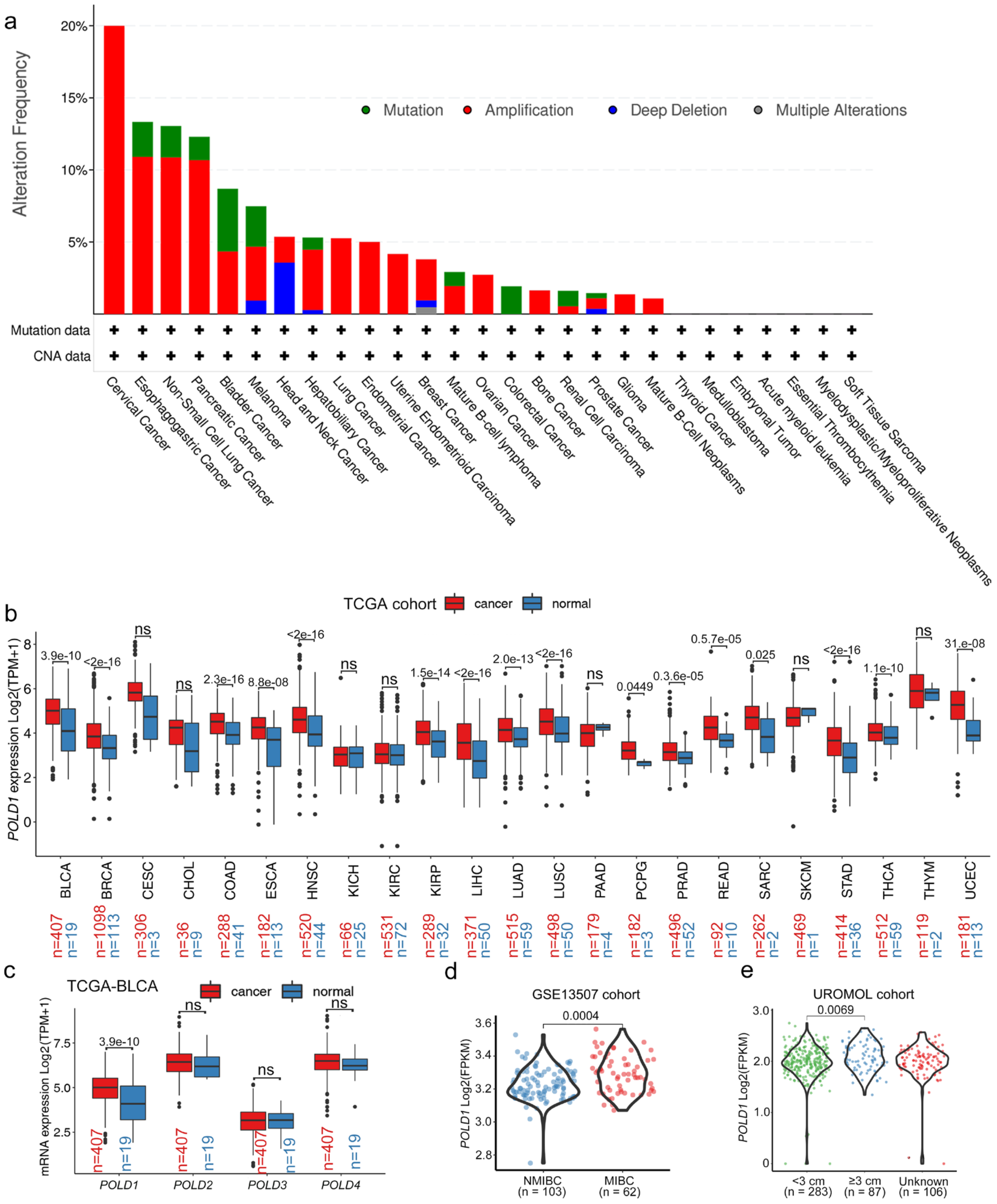

Supplementary Figure 1. *POLD1* is amplified and upregulated in pan-cancer. Related to Figure 1.

a. *POLD1* is commonly amplified in pan-cancer (the red represents the amplification), including BLCA. The X-axis represents each cancer, and the Y-axis represents the frequency of mutations and copy number variations in *POLD1*. The data were downloaded from the cBioPortal website (*https://www.cbioportal.org/*). **b.** The mRNA expression distribution of *POLD1* in pan-cancer from TCGA database (*https://portal.gdc.cancer.gov/*), the X-axis represents tumor names including BLCA, and the Y-axis represents the expression of *POLD1* ($\log_2$(TPM+1)), the red represents the distribution of *POLD1* in tumor tissue and the blue represents normal tissue. **c.** The mRNA level distribution of four subunits (*POLD1*, *POLD2*, *POLD3*, and *POLD4*) of DNA polymerase δ in TCGA-BLCA, the Y-axis represents the expression of *POLD1*

(log_2(TPM+1)). **(b-c)** The center line indicates the median, bounds of box = 25th and 75th percentiles; bars = 10th and 90th percentiles; whiskers = min to max. **d.** The mRNA distribution of *POLD1* (log_2(PFKM)) in the NMIBC and MIBC subtypes in the GSE13507 cohort**. e.** Distribution of *POLD1* mRNA levels at different tumor sizes ($<$ 3 cm and $\geq$ 3 cm) in the UROMOL cohort. Statistical significance was determined by two-tailed Wilcoxon rank-sum test. The n number represents n biologically independent patient samples in each group. Exact n values are marked in **(b-e)**. ns: not significant. BLCA: Bladder Urothelial Carcinoma; BRCA: Breast invasive carcinoma; CESC: Cervical squamous cell carcinoma and endocervical adenocarcinoma; CHOL: Cholangiocarcinoma; COAD: Colon adenocarcinoma; ESCA: Esophageal carcinoma; HNSC: Head and Neck squamous cell carcinoma; KICH: Kidney Chromophobe; KIRC: Kidney renal clear cell carcinoma; KIRP: Kidney renal papillary cell carcinoma; LIHC: Liver hepatocellular carcinoma; LUAD: Lung adenocarcinoma; LUSC: Lung squamous cell carcinoma; PAAD: Pancreatic adenocarcinoma; PCPG: Pheochromocytoma and Paraganglioma; PRAD: Prostate adenocarcinoma; READ: Rectum adenocarcinoma; SARC: Sarcomav; SKCM: Skin Cutaneous Melanoma; STAD: Stomach adenocarcinoma; THCA: Thyroid carcinoma; THYM: Thymoma; UCEC: Uterine Corpus Endometrial Carcinoma. NMIBC: non-muscular invasive bladder cancer; MIBC: muscular invasive bladder cancer; TPM: Transcripts Per Kilobase Million; FPKM: Fragments Per Kilobase Million; ns: not significant.

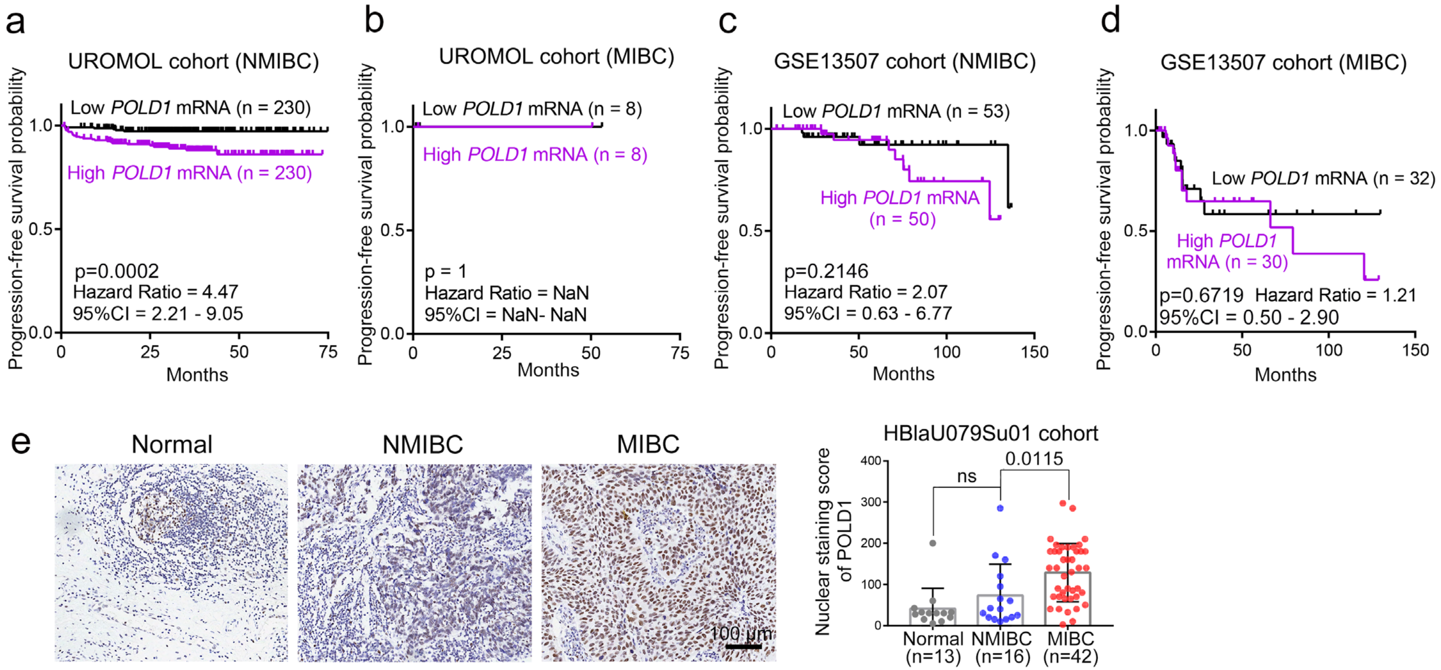

Supplementary Figure 2. POLD1 is associated with prognosis and malignancy of BLCA. Related to Figure 1.

The prognostic curve (progression-free survival) of different *POLD1* mRNA levels in the two subtypes of **a**. NMIBC and **b**. MIBC in the UROMOL cohort was analyzed. The prognostic curve (progression-free survival) of different *POLD1* mRNA levels in the two subtypes of **c**. NMIBC and **d**. MIBC in the GSE13507 cohort was analyzed. The patients were divided into high *POLD1* mRNA level group and low *POLD1* mRNA level group according to the median *POLD1* expression. Statistical significance was determined by two-tailed log-rank test of Kaplan-Meier analysis. The n number represents n biologically independent patient samples in each group. Exact n values are marked in **(a-d)**. **e**. Representative pictures (left panel) and statistical figures (right panel) of immunohistochemistry staining analysis of POLD1 protein level in different type of organization (Normal: n = 13 tissue samples; NMIBC: n = 16 tissue samples; MIBC: n = 42 tissue samples) in HBlaU079Su01 cohort. POLD1 nuclear staining score = The intensity of nuclear staining×Staining positive area. Data are mean ± SD. Statistical significance was determined by two-tailed Student's T-test. NMIBC: non-muscular invasive bladder cancer; MIBC: muscular invasive bladder cancer; CI: Confidence Interval; NaN: Not a Number (Since there was no progression event in both groups of patients, HR and CI could not be calculated here, and NaN was automatically

given by R software); ns: not significant.

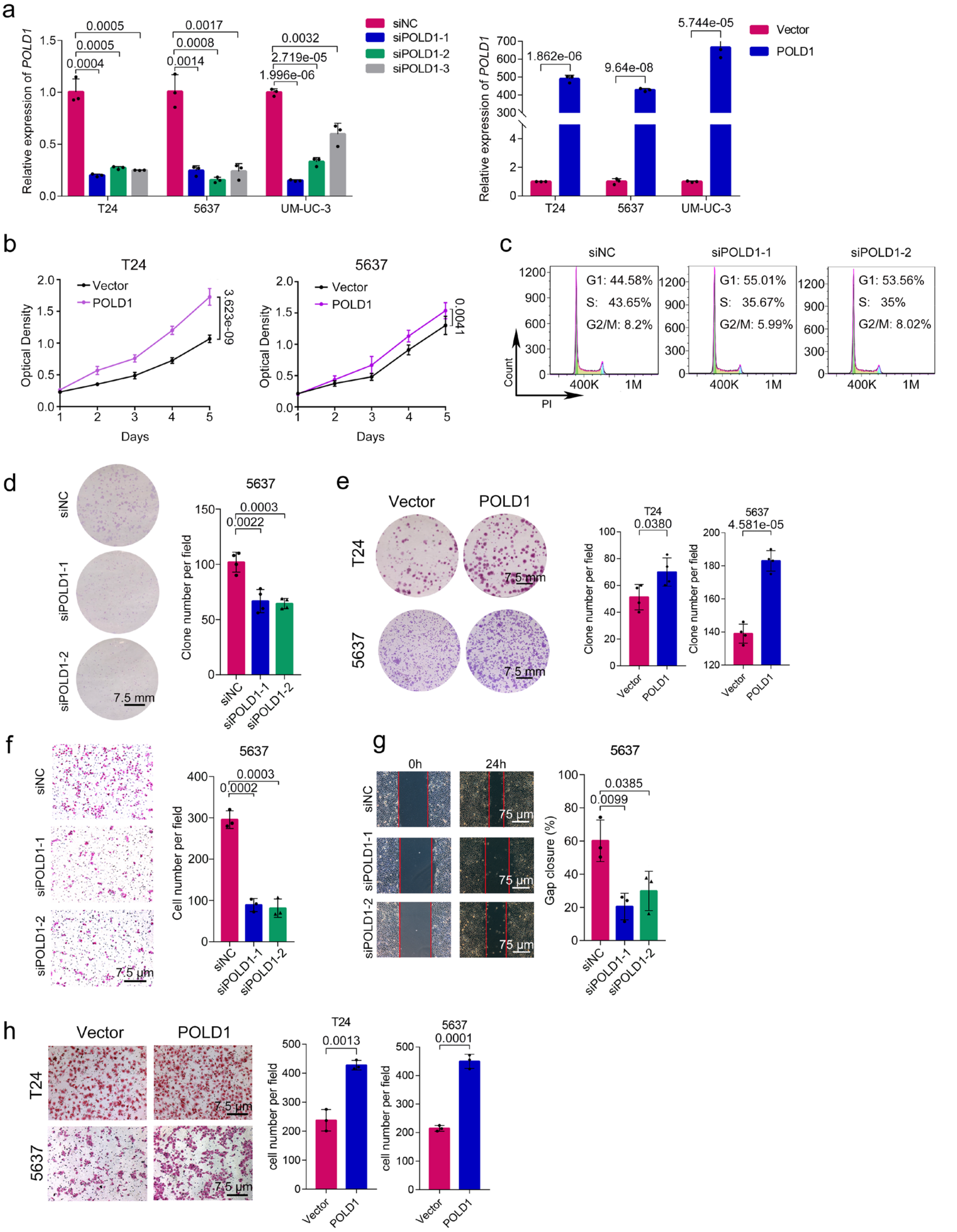

Supplementary Figure 3. POLD1 promotes BLCA proliferation and metastasis. Related to Figure 2.

a. Validation of knockdown efficiency of three *POLD1*-specific siRNA by qPCR assays in T24, 5637 and UM-UC-3 cells (left panel). Validation of GFP-POLD1 overexpression efficiency in T24, 5637 and UM-UC-3 cells by qPCR assays (right panel). Graph shows mean ± SD, n = 3 biologically independent experiments in each group. **b.** The cell proliferation curve of T24 and 5637 cells with POLD1 overexpression. Graph shows mean ± SD, n = 8 biologically independent experiments in each group. **c.** Flow cytometric analysis of DNA content for cell-cycle progression from propidium iodide (PI, 100 μg/mL) staining of T24 cells with or without POLD1 overexpression. **d.** Representative images (left panel) and statistical graph (right panel)

of clone formation assays from the indicated groups with *POLD1* depletion in 5637 cells (n = 4 biologically independent experiments in each group). **e.** Representative images (left panel) and statistical graph (right panel) of clone formation assays from the indicated groups with POLD1 overexpression in T24 and 5637 cells (n = 4 biologically independent experiments in each group). **f.** Representative images (left panel) and cell numbers (right panel) of transwell assays from the indicated groups with or without *POLD1* knockdown in 5637 cells (n = 3 biologically independent experiments in each group). **g.** Representative images (left panel) and statistical results (right panel) of wound healing assay in 5637 cells with or without *POLD1* depletion (n = 3 biologically independent experiments in each group). Gap closure (%) = (0 h distance - 24 h distance) / 0 h distance 100%. The "distance" here refers to the shortest distance between the red lines in the diagram. **h.** Representative images (left panel) and cell numbers (right panel) of transwell assays from the indicated groups with or without POLD1 overexpression in T24 and 5637 cells (n = 3 biologically independent experiments in each group). Data are mean ± SD. Statistical significance was determined by two-tailed Student's T-test.

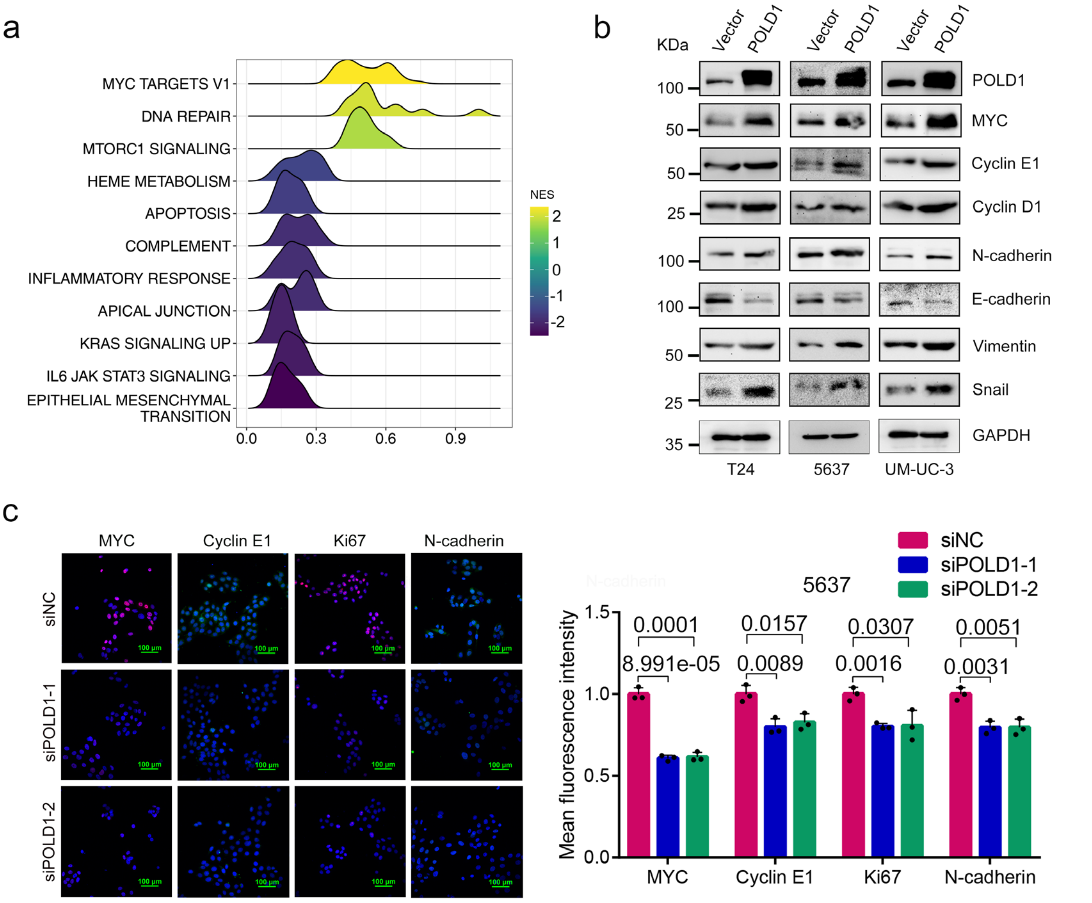

Supplementary Figure 4. The abnormal expression of POLD1 leads to the disturbance of cell cycle and EMT process. Related to Figure 2.

a. GSEA of genes that were both positively correlated with *POLD1* (TGCA-BLCA, the gene set with significant positive correlation with *POLD1* in TCGA-BLCA cohort, $p < 0.05$ and Pearson correlation coefficient > 0) and positively regulated by *POLD1* (the gene set was significantly down-regulated after *POLD1* was knocked down in 5637 cells, $p < 0.05$). The GSEA here was performed by the R package “clusterProfiler”. The X-axis showed $\log_2$ times of the change in the expression of the core enriched genes in the enrichment pathway, with a positive value indicating up-regulated expression and a negative value indicating down-regulated expression. **b.** Immunoblot of cell cycle- (Cyclin E1, Cyclin D1) and EMT- (N-cadherin, E-cadherin, Vimentin and Snail) related protein with or without POLD1 overexpression in T24, 5637 and UM-UC-3. GAPDH was used as loading control. **c.** Representative images of immunofluorescence analysis (left panel) and statistics results (right panel) of the effect of *POLD1* knockdown on MYC, Cyclin E1, Ki67 and N-cadherin in 5637 cells. The average fluorescence intensity was calculated by Image J software. (Graph shows mean ± SD, n = 3 biologically independent experiments in each group). Statistical significance was determined by two-tailed Fisher’s exact test (**a**) and two-tailed Student’s T-test (**c**). NES: Normalized Enrichment Score.

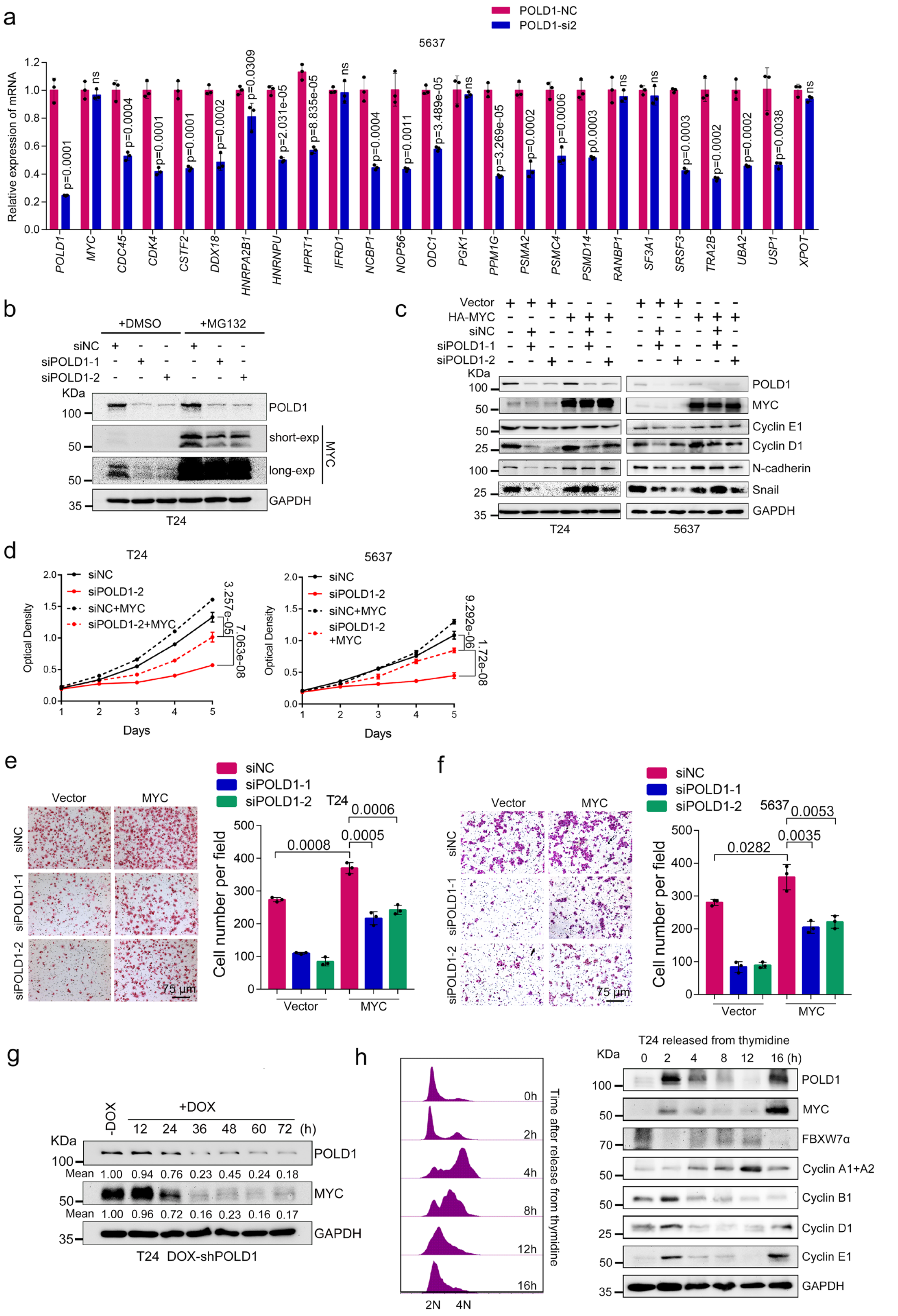

Supplementary Figure 5. POLD1 promotes BLCA proliferation and metastasis by stabilizing MYC. Related to Figure 4.

a. qPCR test to verify the differential expression of MYC target genes after *POLD1* depletion in RNA-seq assay (corresponding to the gene set in Fig. 4b). Graph shows mean ± SD, n = 3 biologically independent experiments in each group. **b.** The changes of MYC were analyzed by Western blot after administration of MG132 in T24 cells

with *POLD1* knocked down. **c.** Western blot was used to detect the changes of Cyclin E1, Cyclin D1, N-cadherin and Snail after MYC overexpression in T24 and 5637 cells with *POLD1* depletion. **d.** Proliferation curves of MYC overexpression in T24 and 5637 cells after *POLD1* knockdown (Graph shows mean ± SD, n = 6 biologically independent experiments in each group). **e-f.** Representative images and cell numbers of transwell assays from the indicated groups with or without overexpression MYC in T24 (**e**) and 5637 (**f**) cells with *POLD1* knockdown (Data are mean ± SD, n = 3 biologically independent experiments in each group). **g.** Representative images of Western blot analysis of the expression changes of POLD1 and MYC after *POLD1* knockdown induced by DOX at gradient time point (n = 3 biologically independent experiments in each group). The protein level of POLD1 and MYC was quantified by Image J software and standardized with GAPDH. **h.** The protein abundance of POLD1, MYC and FBXW7α fluctuates during cell cycle progression. T24 cells synchronized in late G1/S phase by double thymidine (2 mM) following by releasing back into the cell cycle. Left panel: flow cytometry of cell cycle changes at different time points after the cell cycle restart. Right panel: Western blot analysis of protein expression of POLD1, MYC, and FBXW7α at different time points after cell cycle restart. GAPDH was used as loading control. Statistical significance was determined by two-tailed Student's T-test (**a, d-f**). short-exp: short exposure; long-exp: long exposure; ns: not significant.

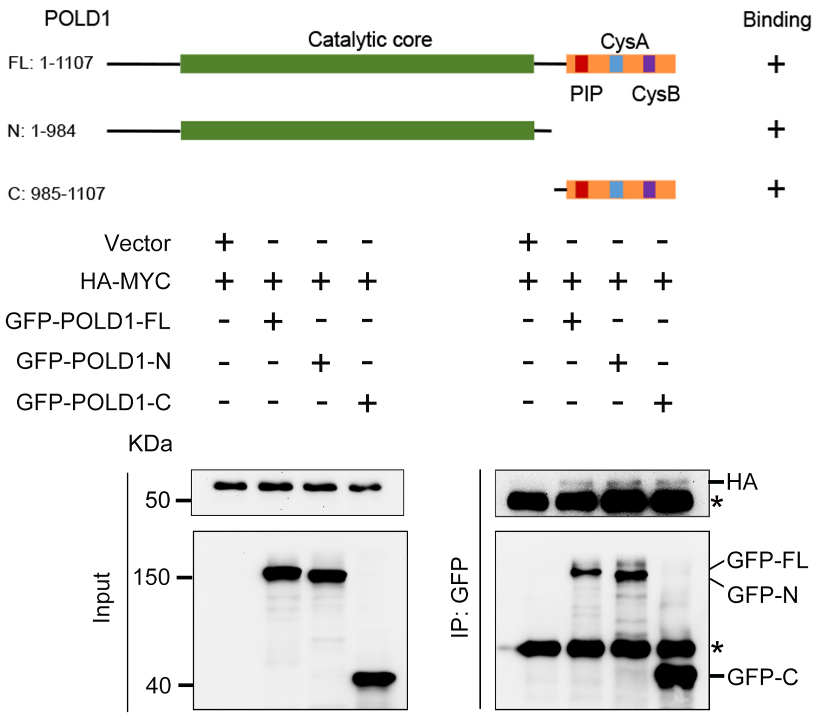

Supplementary Figure 6. MYC can bind to the N-terminal and C-terminal domains of POLD1. Related to Figure 4.

Schematic diagram of various POLD1 truncations in the binding assays (top panel). Western blot analysis of GFP-POLD1, HA-MYC after GFP-IP in 293T cells with overexpression fragment GPF-POLD1 and full-length HA-MYC (bottom panel). The input was 10% of the extract used for the IP. * represents the heavy chain. PIP: PCNA-interacting protein motif; CysA: The cysteine-rich metal-binding motif A; CysB: The cysteine-rich metal-binding motif B.

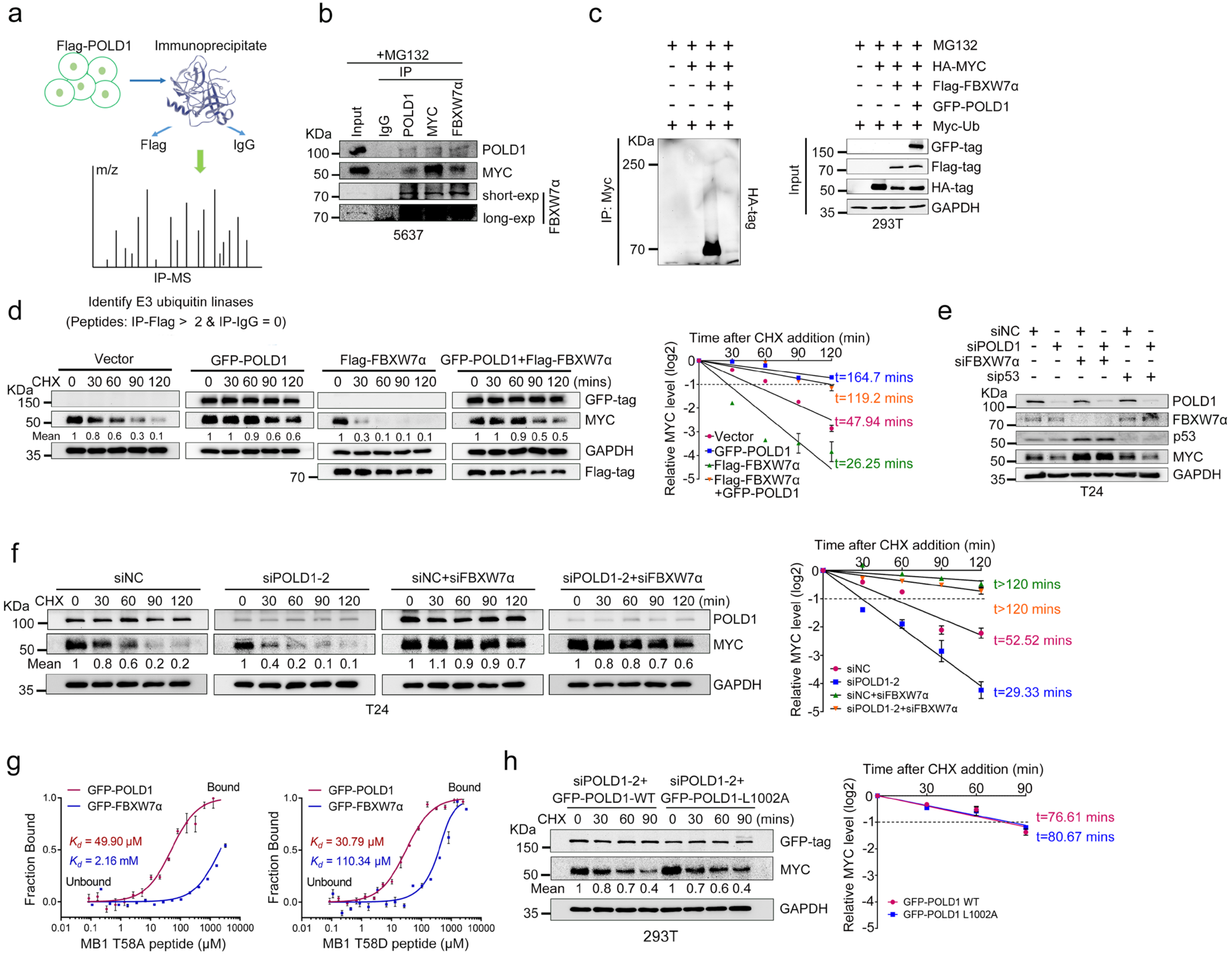
a
Flag-POLD1
Immunoprecipitate
Flag
IgG
m/z
IP-MS
Identify E3 ubiquitin linases
(Peptides: IP-Flag > 2 & IP-IgG = 0)
b
+MG132
IP
Input
IgG
POLD1
MYC
FBXW7α
KDa
POLD1
MYC
short-exp
long-exp
FBXW7α
5637
c
MG132
HA-MYC
Flag-FBXW7α
GFP-POLD1
Myc-Ub
IP: Myc
HA-tag
Input
GFP-tag
Flag-tag
HA-tag
GAPDH
293T
d
Vector
GFP-POLD1
Flag-FBXW7α
GFP-POLD1+Flag-FBXW7α
CHX 0 30 60 90 120 (mins)
GFP-tag
MYC
Mean
GAPDH
Flag-tag
Time after CHX addition (min)
Relative MYC level (log2)
t=164.7 mins
t=119.2 mins
t=47.94 mins
t=26.25 mins
Vector
GFP-POLD1
Flag-FBXW7α
Flag-FBXW7α +GFP-POLD1
e
siNC
siPOLD1
siFBXW7α
sip53
POLD1
FBXW7α
p53
MYC
GAPDH
T24
f
siNC
siPOLD1-2
siNC+siFBXW7α
siPOLD1-2+siFBXW7α
CHX 0 30 60 90 120 (min)
POLD1
MYC
Mean
GAPDH
T24
Time after CHX addition (min)
Relative MYC level (log2)
t>120 mins
t>120 mins
t=52.52 mins
t=29.33 mins
g
GFP-POLD1
GFP-FBXW7α
Bound
Unbound
K_d = 49.90 μM
K_d = 2.16 mM
Fraction Bound
MB1 T58A peptide (μM)
K_d = 30.79 μM
K_d = 110.34 μM
MB1 T58D peptide (μM)
h
siPOLD1-2+ GFP-POLD1-WT
siPOLD1-2+ GFP-POLD1-L1002A
CHX 0 30 60 90 0 30 60 90 (mins)
GFP-tag
MYC
Mean
GAPDH
293T
Time after CHX addition (min)
Relative MYC level (log2)
t=76.61 mins
t=80.67 mins
GFP-POLD1 WT
GFP-POLD1 L1002A

Supplementary Figure 7. POLD1 competes with FBXW7α to bind MYC in a manner independent of DNA polymerase activity, thereby stabilizing MYC. Related to Figure 6.

a. Schematic diagram of screening E3 ubiquitin ligases that interact with POLD1. Flag-POLD1 was overexpressed in 293T cells and then lysed. The IgG and Flag-IP assays were carried out on the lysate respectively, and then the interacting proteins were detected by mass spectrometry. The screening condition was set as unique peptides > 2 and IgG results were negative. **b.** Western blot analysis of POLD1, MYC and FBXW7α after IgG-IP, POLD1-IP, MYC-IP or FBXW7α-IP in 5637 cells. MYC was used as a positive control for FBXW7α endogenous interaction. The input was 10% of the extract used for the endogenous IP. All samples were added with MG132 (10 μM) for 6 h before harvesting cells. **c.** Western blot of ubiquitination of HA-MYC after Myc-Ub-IP under denaturing conditions in 293T cells expressing GFP-POLD1, HA-MYC, Flag-FBXW7α and Myc-Ub. The cells of all samples were incubated with MG132 (10 μM) for 6 h before harvest. **d.** Representative images of the effect of Vector or GFP-POLD1 or Flag-FBXW7α or GFP-POLD1 plus Flag-FBXW7α overexpression on MYC degradation in 293T cells were incubated with CHX (50 μg/mL) for the indicated times (left panel). Statistical diagram of protein half-life assays (right panel). **e.** Western blot analysis of POLD1, MYC, FBXW7α and p53 after *FBXW7α* or *p53* depletion in T24 cells with *POLD1* knockdown. **f.** Representative images of Western blot analysis (left panel) and statistics results (right panel) of MYC half-life after depletion of *FBXW7α* in T24 cells with *POLD1* knockdown. **g.** MST analysis was used to measure the binding affinity of lysates of overexpressed GFP-POLD1 or GFP-FBXW7α in 293T cells and the MYC-MB1 T58A (left panel) and MYC-MB1 T58D (right panel) peptide. **h.** Representative images of Western blot analysis (left panel) and statistics results (right panel) of MYC half-life after overexpression GFP-POLD1 WT or GFP-POLD1 L1002A in 293T cells with *POLD1* knockdown. The cells were incubated with CHX (50 μg/mL) for the indicated times. Data are mean ± SD, n = 3 biologically independent experiments in each group (**d, f-h**). GAPDH was used as loading control (**c-f**, **h**). All proteins in this figure were quantified by Image J software. short-exp: short exposure; long-exp: long exposure.

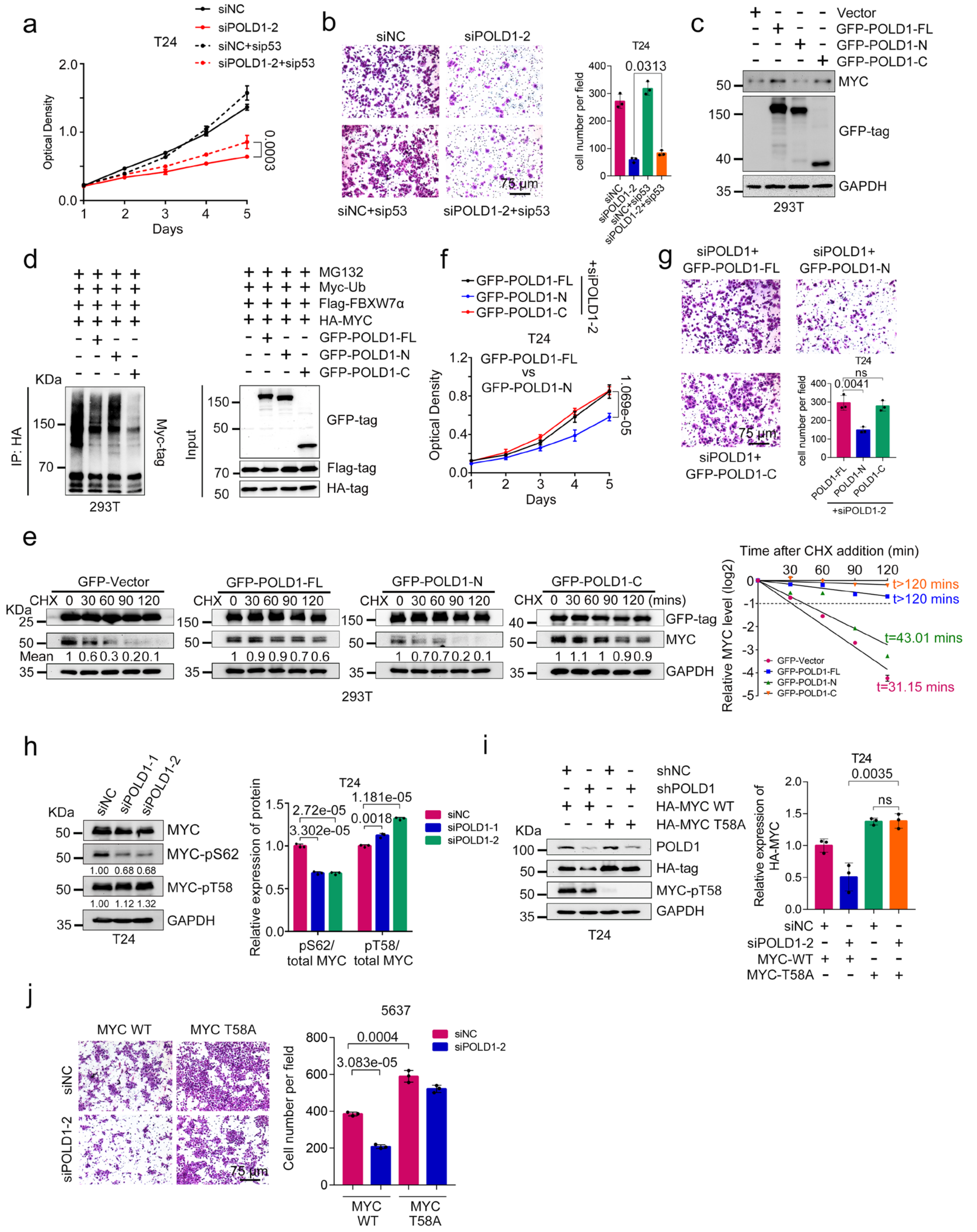

Supplementary Figure 8. The C-terminal domain of POLD1 plays a key role in stabilizing MYC. Related to Figure 6.

a. The cell proliferation curve of *p53* deletion in T24 cells with *POLD1* knockdown. Graph shows mean ± SD, n = 6 biologically independent experiments in each group. **b.** Representative images (left panel) and cell numbers (right panel) of transwell assays from the indicated groups with or without *p53* deletion in T24 cells with *POLD1* knockdown (n = 3 biologically independent experiments in each group). **c.** Western blot of MYC after full-length and fragment GFP-POLD1 was overexpression in 293T cells. **d.** Western blot of Myc-Ub after HA-IP under denaturing conditions in 293T cells expressing full-length and fragment GFP-POLD1, HA-MYC, Flag-FBXW7α and Myc-

Ub. The cells of all samples were incubated with MG132 (10 μM) for 6 h before harvest. **e.** Representative images of Western blot analysis (left panel) and statistics results (right panel) of MYC half-life after full-length and fragment GFP-POLD1 was overexpression in 293T cells. The cells were incubated with CHX (50 μg/mL) for the indicated times (n = 3 biologically independent experiments in each group). **f.** The cell proliferation curve of overexpressed full-length and fragment GFP-POLD1 in T24 cells with *POLD1* knockdown. Graph shows mean ± SD, n = 6 biologically independent experiments in each group. **g.** Representative images (left panel) and cell numbers (right panel) of transwell assays after full-length and fragment GFP-POLD1 overexpressioned in T24 cells with *POLD1* knockdown (n = 3 biologically independent experiments in each group). **h.** Representative images of Western blot (left panel) and statistics results (right panel) of totol MYC, MYC of phosphorylated serine 62 (pS62) and MYC of phosphorylated threonine-58 (pT58) after *POLD1* deleption in T24 cells (n = 3 biologically independent experiments in each group). **i.** Representative images of Western blot (left panel) and statistics results (right panel) of POLD1, totol MYC, and MYC of phosphorylated threonine-58 (pT58) after WT or T58A HA-MYC overexpressioned in T24 cells with *POLD1* deleption (n = 3 biologically independent experiments in each group). **j.** Representative images (left panel) and cell numbers (right panel) of transwell assays with or without WT or T58A HA-MYC overexpressioned in 5637 cells with *POLD1* knockdown (n = 3 biologically independent experiments in each group). Graph shows mean ± SD. All proteins in this figure were quantified by Image J software. GAPDH was used as loading control (**c**, **e**, **h**-**i**). Statistical significance was determined by two-tailed Student's T-test (**a**-**b**, **f**-**j**). ns: not significant.

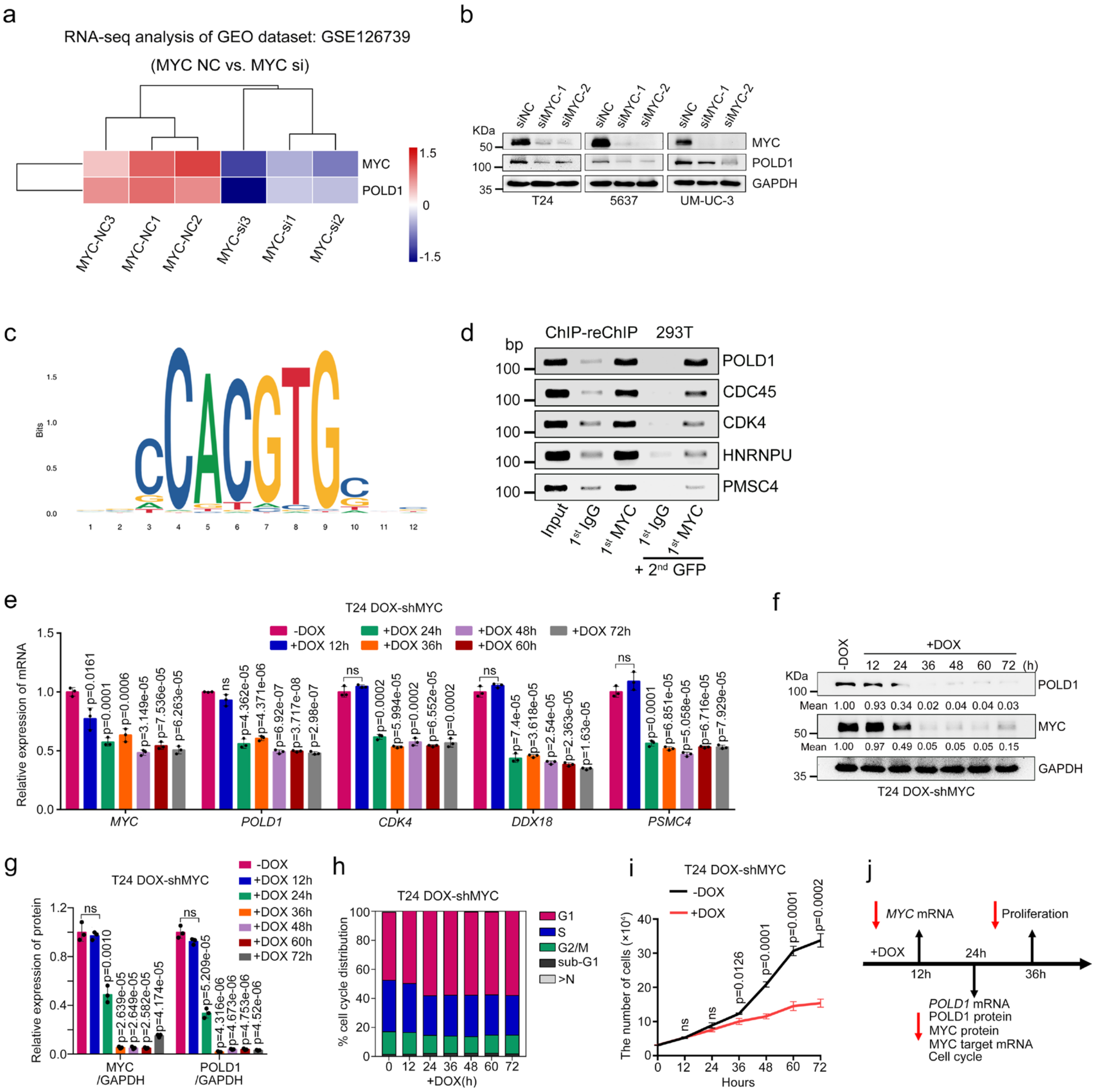

Supplementary Figure 9. *POLD1* is the target gene of MYC transcriptional regulation, and can form complexes with MYC to enhance the transcriptional activity of MYC. Related to Figure 7.

a. A heatmap of *POLD1* and *MYC* upon *MYC* knockdown in lymphoma Jurkat cells (n = 3 per group). **b.** Western blot of POLD1 and MYC after *MYC* deplection in T24, 5637 and UM-UC-3 cells. **c.** The motif sequence of MYC provided by the JASPAR database (*https://jaspar.genereg.net/*). **d.** The POLD1/MYC complex was found on *POLD1*, *CDC45*, *CDK4*, etc. promoters. ChIP and reChIP assays were performed in 293T cells with the indicated antibodies. **e.** qPCR analysis of *MYC* and its target genes in DOX-induced *shMYC* T24 cells. We harvested the cells at the specified time after adding DOX. **f.** Representative images of Western blot analysis and **g.** statistical graph of the expression changes of POLD1 and MYC after *MYC* knockdown induced by DOX at gradient time point. **h.** Cell cycle and **i.** proliferation analysis after *MYC* knockdown induced by DOX at gradient time point. **j.** Timeline of all assay results of DOX-induced *MYC* knockdown in this section. Graph shows mean ± SD, n = 3 biologically independent experiments in each group (**e, g, i**). Statistical significance was determined by two-tailed Student's T-test (**e, g, i**). ns: not significant.

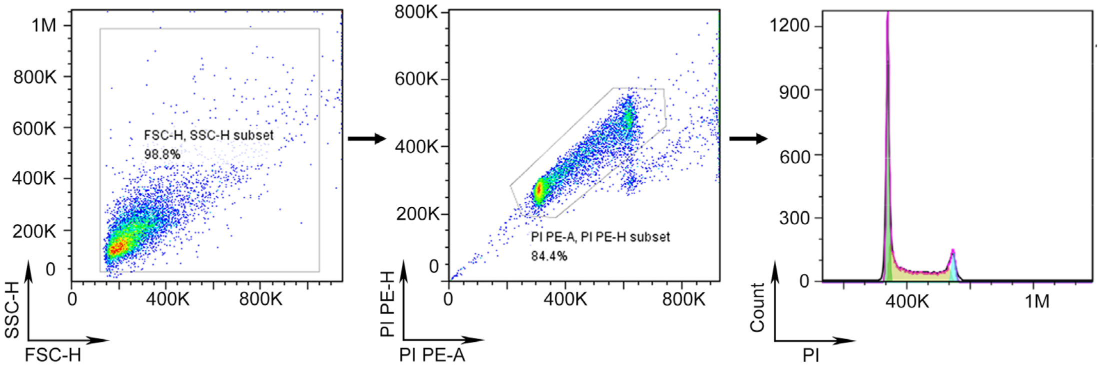

Supplementary Figure 10. Gating strategy for flow analysis.

This gated strategy was used in all flow cytometric cell cycle correlation analyses in this study (Related to Fig. 2b, 4i, and Supplementary Fig. 3c, 5h, 9h). The cell population was identified using FSC and SSC (left pannel), this cell population gate is then placed on PI PE-A and PI PE-H plot (middle pannel), finally, the cell cycle curve was fitted (right pannel).

Supplementary Tables

Supplementary Table 1. POLD1 related GSEA analysis.

Description	setSize	enrichmentScore	NES	pvalue	p.adjust	qvalues	rank
MYC_TARGETS_V1	44	0.571823958	2.35538	3.61E-08	1.05E-06	6.08E-07	360
DNA_REPAIR	19	0.589086165	2.021741	0.000207	0.0015	0.000871	237
MTORC1_SIGNALING	49	0.414334099	1.736648	0.001654	0.007995	0.004643	233
HEME_METABOLISM	24	-0.277905838	-1.49354	0.011575	0.033567	0.019494	607
APOPTOSIS	18	-0.334238928	-1.60731	0.014404	0.037974	0.022054	423
COMPLEMENT	21	-0.349614234	-1.86622	0.005598	0.018039	0.010476	582
INFLAMMATORY_RESPONSE	31	-0.300028389	-1.86804	0.001966	0.008146	0.004731	506
APICAL_JUNCTION	15	-0.413718017	-1.89225	0.004276	0.015502	0.009003	511
KRAS_SIGNALING_UP	32	-0.330648923	-2.0868	0.000161	0.0015	0.000871	253
IL6_JAK_STAT3_SIGNALING	15	-0.474612666	-2.17077	0.000802	0.004652	0.002701	423
EPITHELIAL_MESENCHYMAL_TRANSITION	30	-0.404172543	-2.47338	2.23E-05	0.000324	0.000188	391

NES: Normalized Enrichment Score. Statistical significance of the GSEA analysis was determined by two-tailed Fisher's exact test.

Supplementary Table 2. POLD1 related GO analysis.

ONTOLOGY	ID	Description	pvalue	p.adjust	qvalue	Count
BP	GO:0042060	wound healing	0.000804298	0.0264204	0.02293355	38
BP	GO:0034599	cellular response to oxidative stress	7.99E-06	0.0010429	0.00090522	34
BP	GO:0045740	positive regulation of DNA replication	4.31E-06	0.0006616	0.00057431	11
BP	GO:0006275	regulation of DNA replication	1.03E-05	0.0012503	0.00108533	18
BP	GO:0006261	DNA-dependent DNA replication	3.73E-06	0.0005947	0.00051625	23
BP	GO:2000278	regulation of DNA biosynthetic process	2.22E-06	0.0004148	0.00036006	19
BP	GO:0071496	cellular response to external stimulus	4.26E-09	5.56E-06	4.83E-06	44
BP	GO:0006260	DNA replication	2.92E-08	2.54E-05	2.21E-05	37
BP	GO:0044786	cell cycle DNA replication	0.001111072	0.0329723	0.02862075	8
MF	GO:0003688	DNA replication origin binding	0.001277548	0.0445722	0.03934748	5
MF	GO:0051087	chaperone binding	0.000227239	0.0125917	0.01111571	16
MF	GO:0051787	misfolded protein binding	0.000119592	0.0086659	0.00765004	8
MF	GO:0044389	ubiquitin-like protein ligase binding	5.93E-05	0.0059371	0.00524118	35
MF	GO:0001671	ATPase activator activity	6.82E-05	0.0059371	0.00524118	8
MF	GO:0031625	ubiquitin protein ligase binding	1.61E-05	0.0021574	0.00190446	35
MF	GO:0004529	exodeoxyribonuclease activity	1.83E-05	0.0021574	0.00190446	8
MF	GO:0045296	cadherin binding	3.60E-07	0.0001132	9.99E-05	42
MF	GO:0003697	single-stranded DNA binding	3.08E-06	0.0007254	0.0006404	21
CC	GO:0005925	focal adhesion	1.05E-06	0.0002823	0.00024384	46
CC	GO:0030055	cell-substrate junction	1.66E-06	0.0002823	0.00024384	46
CC	GO:0030684	preribosome	1.84E-06	0.0002823	0.00024384	16
CC	GO:0005635	nuclear envelope	1.99E-06	0.0002823	0.00024384	49
CC	GO:0042470	melanosome	2.76E-06	0.0002823	0.00024384	19
CC	GO:0048770	pigment granule	2.76E-06	0.0002823	0.00024384	19
CC	GO:1904813	ficolin-1-rich granule lumen	5.25E-06	0.0004601	0.00039744	20
CC	GO:0101031	chaperone complex	9.68E-06	0.0006693	0.00057813	9
CC	GO:0101002	ficolin-1-rich granule	9.83E-06	0.0006693	0.00057813	25

BP: Biological Process; MF: Molecular Function; CC: Cellular Component. Statistical significance of the GO analysis was determined by two-tailed Fisher's exact test.

Supplementary Table 3. Details of antibodies.

Antibody	Identifier	Source	Dilution or amount
anti-GST-Tag	MY1903	MerryBio	IP/1 μg WB/1:1000
anti-His-Tag	66005-1-Ig	Proteintech	IP/1 μg WB/1:1000
anti-HA-Tag	TA180128	Origene	IP/1 μg WB/1:1000
anti-GFP-Tag	SC-9996	Santa Cruz	IP/1 μg WB/1:1000
anti-GFP-Tag	ab290	Abcam	IP/1 μg WB/1:1000 ChIP/8μg
anti-Flag-Tag	F1804	Sigma	IP/1 μg WB/1:1000
anti-Myc-Tag	AE010	Abclonal	IP/1 μg WB/1:1000
anti-POLD1	SC-17776	Santa Cruz	WB/1:200 IHC/1:50 IF/1:50
anti-POLD1	ab264345	Abcam	IP/1 μg WB/1:1000
anti-MYC	18583	Cell Signaling Technology	IP/1 μg WB/1:1000 IF/1:200
anti-MYC	ab32072	Abcam	WB/1:1000 ChIP/8 μg IHC/1:100
anti-MYC-pT58	46650	Cell Signaling Technology	WB/1:1000
anti-MYC-pS62	13748	Cell Signaling Technology	WB/1:1000
anti-FBXW7	ab109617	Abcam	IP/1 μg WB/1:1000 IF/1:200
anti-Cyclin A1+A2	ab185619	Abcam	WB/1:1000
anti-Cyclin B1	4135S	Cell Signaling Technology	WB/1:1000
anti-Cyclin E1	SC-377100	Santa Cruz	WB/1:200 IF/1:50
anti-Cyclin D1	SC-753	Santa Cruz	WB/1:200
anti-N-cadherin	13116S	Cell Signaling Technology	WB/1:1000
anti-E-cadherin	3195S	Cell Signaling Technology	WB/1:1000
anti-Vimentin	5741S	Cell Signaling Technology	WB/1:1000
anti-Snail	3879S	Cell Signaling Technology	WB/1:1000
anti-MAX	SC-8011	Santa Cruz	IP/1 μg WB/1:200
anti-Ubiquitin	ab7254	Abcam	WB/1:1000
anti-p53	10442-1-AP	Proteintech	WB/1:1000
anti-GAPDH	SC-365062	Santa Cruz	WB/1:200
anti-Ki67	ab16667	Abcam	IHC/1:200 IF/1:200
anti-MYC	10828-1-AP	Proteintech	IF 1:100
anti-N-cadherin	66219-1-Ig	Proteintech	IF 1:200

Supplementary Table 4. Primers used in qPCR assays.

qPCR assay:

Gene Symbol	Forward	Reverse
POLD1	ACCTTCATCCGTATCATGGAC	ATGAAGAGTCCCGGATGTTG
MYC	CTGGTGCTCCATGAGGAGA	CCTGCCTCTTTTCCACAGAA
CDC45	CTTGAAGTTCCCGCCTATGAAG	GCATGGTTTGCTCCACTATCTC
USP1	TCATTTCGGTTGAACAGCTCC	CCCTCAGTGTGTTAAGCAGTC
TRA2B	GCAGTCTAGGCGTTCAAGAGG	CATTGGCACGTTCTTTAGCTTC
PPM1G	GAGCTGCTGACACGCTACG	TTCCCTAGTTGAGTCCTCAGG
RANBP1	AATACAGACGAGTCCAACCATGA	GAACAGTTTTGCCCGCATTTTA
CDK4	ATGGCTACCTCTCGATATGAGC	CATTGGGGACTCTCACACTCT
SRSF3	TGGCAACAAGACGGAATTGGA	CAAAGCCGGGTGGGTTTCTA
NOP56	ACAGGAGGAGTTAGGGTACAAC	GATTGTGGAAGTGCAGACGAA
UBA2	TTCTCCCACATCGACCTGATT	ACCTGTGCCTTTGATCTTCCA
HNRNPA2B1	ATTGATGGGAGAGTAGTTGAGCC	AATTCCGCCAACAAACAGCTT
HNRNPU	GAGCATCCTATGGTGTGTCAAA	TGACCAGCCAATACGAACTTC
CSTF2	CAGCGGTGGATCGTTCTCTAC	AACAACAGGTCCAACCTCAGA
NCBP1	GGAGAGCAACCTAGAAGGCTT	AGGTAATAGGCGTGCAACTGT
HPRT1	CCTGGCGTCGTGATTAGTGAT	AGACGTTCAGTCCTGTCCATAA
PSMD14	GATGGTTGTTGGTTGGTATCACA	AGCTCTCTCCGACAAGGCTT
PSMA2	GAGCGCGGGTACAGCTTTT	ACCACACCATTTGCAGCTTTA
DDX18	ATGTCACACCTGCCGATGAAA	CCCTGAAACTTTAGGTTCCGC
PSMC4	CCGCTGGTCATCGGACAATTT	GCGCACATAATAGTTGGAGCCT
XPOT	CAAGTCTTCGCCTTGCTTTTTG	CCCTTGGATTTAGGTCCACTACT
PGK1	GACCTAATGTCCAAAGCTGAGAA	CAGCAGGTATGCCAGAAGCC
ODC1	GTTTTGCGGATTGCCACTGAT	GCTCTTTCGCCCGTTCCAA
SF3A1	TTCACGAAGCTAGTGGAACAGT	GGTAACACACCTGATCCAAAACT
IFRD1	GAGTGCGAAGACAAGGCAAG	GCAGCGTTCAATGCTATCAGTT
GAPDH	CTGGGCTACACTGAGCACC	AAGTGGTCGTTGAGGGCAATG

ChIP assay:

Gene Symbol	Forward	Reverse
POLD1	CACGAGGTCGTGAAGGTAGC	TCGCCCCCTTGCTGAAAAAT
CDK4	CGTGAGGTAAGTGCAGTCCC	CATAACCAGCTCGCGAAACG
CDC45	AAGAGAAAAGGCCAGGCGAA	GGGGAAATAAATCACGAAAGGGC
HNRNPU	GCCAGTCTCCTCGATGATTCG	CGCTCTCTTCGGGATACACG
PSMC4	ACGATCAATCCCTTCTGTGG	CCAACTGCTCTTCGCTTTCT

Supplementary Table 5. Clinicopathological characteristics of TCGA cohort.

			POLD1 expression			
	**Variables**	**Total (n=398)**	**High (n=199)**	**Low (n=199)**	**p value**	**Statistics method (two-tailed)**
Gender (%)	Female	108 (27.14)	52 (26.13)	56 (28.14)	0.7352	Chi-square
	Male	290 (72.86)	147 (73.87)	143 (71.86)		
Age (year) (%)	< 65	149 (37.53)	72 (36.18)	77 (38.89)	0.6502	Chi-square
	>= 65	248 (62.47)	127 (63.82)	121 (61.11)		
Subtype (%)	MIBC	392 (98.49)	197 (98.99)	195 (97.99)	0.6808	Chi-square
	NMIBC	6 (1.51)	2 (1.01)	4 (2.01)		
Stage (%)	I	3 (0.75)	0 (0.00)	3 (1.51)	0.0714	Fisher's exact
	II	108 (27.14)	48 (24.12)	60 (30.15)		
	III	147 (36.93)	83 (41.71)	64 (32.16)		
	IV	138 (34.67)	67 (33.67)	71 (35.68)		
Grade (%)	High	376 (94.95)	195 (98.48)	181 (91.41)	**0.002**	Fisher's exact
	Low	20 (5.05)	3 (1.52)	17 (8.59)		
T (%)	Tx	1 (0.25)	0 (0.00)	1 (0.50)	0.1227	Fisher's exact
	T0	1 (0.25)	1 (0.50)	0 (0.00)		
	T1	4 (1.01)	1 (0.50)	3 (1.51)		
	T2	124 (31.16)	54 (27.14)	70 (35.18)		
	T3	206 (51.76)	114 (57.29)	92 (46.23)		
	T4	62 (15.58)	29 (14.57)	33 (16.58)		
M (%)	Mx	195 (49.12)	111 (56.06)	84 (42.21)	**0.019**	Fisher's exact
	M0	194 (48.87)	83 (41.92)	111 (55.78)		
	M1	8 (2.02)	4 (2.02)	4 (2.01)		
N (%)	NX	27 (6.78)	10 (5.03)	17 (8.54)	**0.0123**	Fisher's exact
	N0	236 (59.30)	123 (61.81)	113 (56.78)		
	N1	48 (12.06)	31 (15.58)	17 (8.54)		
	N2	80 (20.10)	30 (15.08)	50 (25.13)		
	N3	7 (1.76)	5 (2.51)	2 (1.01)		

POLD1 expression group: The median of the POLD1 expression was cutoff value.

NMIBC: non-muscular invasive bladder cancer.

MIBC: muscular invasive bladder cancer.

Statistical significance was determined by two-tailed Chi-square or two-tailed Fisher's exact test. No adjustments were made for multiple comparisons.

Supplementary Table 6. Clinicopathological characteristics of UROMOL cohort.

			POLD1 expression			
	**Variables**	**Total (n=476)**	**High (n=238)**	**Low (n=238)**	**p value**	**Statistics method (two-tailed)**
Gender (%)	Female	109 (22.90)	56 (23.53)	53 (22.27)	0.8273	Chi-square
	Male	367 (77.10)	182 (76.47)	185 (77.73)		
Age (year) (%)	< 65	168 (35.29)	74 (31.09)	94 (39.50)	0.0684	Chi-square
	>= 65	308 (64.71)	164 (68.91)	144 (60.50)		
Subtype (%)	MIBC	16 (3.36)	15 (6.30)	1 (0.42)	**0.0009**	Chi-square
	NMIBC	460 (96.64)	223 (93.70)	237 (99.58)		
Stage (%)	CIS	3 (0.63)	3 (1.26)	0 (0.00)	**1.811e-09**	Fisher's exact
	Ta	345 (72.48)	144 (60.50)	201 (84.45)		
	T1	112 (23.53)	76 (31.93)	36 (15.13)		
	T2-4	16 (3.36)	15 (6.30)	1 (0.42)		
Grade (%)	High	192 (40.34)	131 (55.04)	61 (25.63)	**6.595e-11**	Fisher's exact
	Low	277 (58.19)	106 (44.54)	171 (71.85)		
	PUNLMP	7 (1.47)	1 (0.42)	6 (2.52)		
Tumor Size (%)	< 3 cm	283 (59.45)	131 (55.04)	152 (63.87)	0.0588	Chi-square
	>= 3 cm	87 (18.28)	51 (21.43)	36 (15.13)		

POLD1 expression group: The median of the POLD1 expression was cutoff value.

Tumor size: The longest diameter, cm.

NMIBC: non-muscular invasive bladder cancer.

MIBC: muscular invasive bladder cancer.

Statistical significance was determined by two-tailed Chi-square or two-tailed Fisher's exact test. No adjustments were made for multiple comparisons.

Supplementary Table 7. Clinicopathological characteristics of GSE32984 cohort.

			POLD1 expression			
	**Variables**	**Total (n=224)**	**High (n=112)**	**Low (n=112)**	**p value**	**Statistics method (two-tailed)**
Gender (%)	Famale	61 (27.23)	31 (27.68)	30 (26.79)	1	Chi square
	Male	163 (72.77)	81 (72.32)	82 (73.21)		
Age (year) (%)	< 65	79 (35.27)	46 (41.07)	33 (29.46)	0.0933	Chi square
	>= 65	145 (64.73)	66 (58.93)	79 (70.54)		
Subtype (%)	MIBC	51 (22.77)	43 (38.39)	8 (7.14)	**6.046e-08**	Chi square
	NMIBC	173 (77.23)	69 (61.61)	104 (92.86)		
Stage (%)	Ta	110 (49.11)	34 (30.36)	76 (67.86)	**2.274e-09**	Fisher's exact
	T1	63 (28.12)	35 (31.25)	28 (25.00)		
	T2	43 (19.20)	36 (32.14)	7 (6.25)		
	T3	7 (3.12)	6 (5.36)	1 (0.89)		
	T4	1 (0.45)	1 (0.89)	0 (0.00)		
Grade (%)	Gx	2 (0.89)	2 (1.79)	0 (0.00)	**7.491e-10**	Fisher's exact
	G1	45 (20.09)	9 (8.04)	36 (32.14)		
	G2	84 (37.50)	33 (29.46)	51 (45.54)		
	G3	93 (41.52)	68 (60.71)	25 (22.32)		

POLD1 expression group: The median of the POLD1 expression was cutoff value.

NMIBC: non-muscular invasive bladder cancer;

MIBC: muscular invasive bladder cancer.

Statistical significance was determined by two-tailed Chi-square or two-tailed Fisher's exact test. No adjustments were made for multiple comparisons.

Supplementary Table 8. Clinicopathological characteristics of HBlaU079Su01 cohort.

			POLD1 expression			
	**Variables**	**Total (n=63)**	**High (n=26)**	**Low (n=37)**	**p value**	**Statistics method (two-tailed)**
Gender (%)	female	53 (84.13)	23 (88.46)	30 (81.08)	0.6606	Chi-square
	male	10 (15.87)	3 (11.54)	7 (18.92)		
Age (year) (%)	< 65	22 (35.48)	9 (34.62)	13 (36.11)	1	Chi-square
	>= 65	40 (64.52)	17 (65.38)	23 (63.89)		
Subtype (%)	MIBC	42 (66.67)	23 (88.46)	19 (51.35)	**0.0067**	Chi-square
	NMIBC	16 (25.40)	3 (11.54)	13 (35.14)		
AJCC stage (%)	< StageII	11 (17.46)	1 (3.85)	10 (27.03)	**0.0134**	Fisher's exact
	>= StageII	43 (68.25)	23 (88.46)	20 (54.05)		
T (%)	< T2	16 (25.40)	3 (11.54)	13 (35.14)	**0.0049**	Fisher's exact
	>= T2	42 (66.67)	23 (88.46)	19 (51.35)		
N (%)	N >= 1	8 (12.70)	5 (19.23)	3 (8.11)	0.5095	Fisher's exact
	N0	45 (71.43)	17 (65.38)	28 (75.68)		
Tumor Size (%)	< 3 cm	17 (29.82)	8 (32.00)	9 (28.12)	0.9796	Chi-square
	>= 3 cm	40 (70.18)	17 (68.00)	23 (71.88)		

POLD1 staining score = The intensity of nuclear * Positive area

POLD1 staining group: ≤ 120 is low; > 120 is high.

Tumor size: The longest diameter, cm.

NMIBC: non-muscular invasive bladder cancer;

MIBC: muscular invasive bladder cancer.

Statistical significance was determined by two-tailed Chi-square or two-tailed Fisher's exact test. No adjustments were made for multiple comparisons.

Supplementary Table 9. Clinicopathological characteristics of GSE13507 cohort.

			POLD1 expression			
	**Variables**	**Total (n=165)**	**High (n=82)**	**Low (n=83)**	**p value**	**Statistics method (two-tailed)**
Gender (%)	Famale	30 (18.18)	18 (21.95)	12 (14.46)	0.2956	Chi-square
	Male	135 (81.82)	64 (78.05)	71 (85.54)		
Age (year) (%)	>= 65	91 (55.15)	50 (60.98)	41 (49.40)	0.1807	Chi-square
	< 65	74 (44.85)	32 (39.02)	42 (50.60)		
Subtype (%)	NMIBC	103 (62.42)	42 (51.22)	61 (73.49)	**0.0052**	Chi-square
	MIBC	62 (37.58)	40 (48.78)	22 (26.51)		
T (%)	Ta	24 (14.55)	7 (8.54)	17 (20.48)	**0.0043**	Fisher's exact
	T1	80 (48.48)	36 (43.90)	44 (53.01)		
	T2	31 (18.79)	16 (19.51)	15 (18.07)		
	T3	19 (11.52)	16 (19.51)	3 (3.61)		
	T4	11 (6.67)	7 (8.54)	4 (4.82)		
M (%)	M0	158 (95.76)	78 (95.12)	80 (96.39)	0.7197	Fisher's exact
	M1	7 (4.24)	4 (4.88)	3 (3.61)		
N (%)	Nx	1 (0.61)	0 (0.00)	1 (1.20)	**0.0094**	Fisher's exact
	N0	149 (90.30)	69 (84.15)	80 (96.39)		
	N1	8 (4.85)	6 (7.32)	2 (2.41)		
	N2	6 (3.64)	6 (7.32)	0 (0.00)		
	N3	1 (0.61)	1 (1.22)	0 (0.00)		
Grade (%)	High	60 (36.36)	44 (53.66)	16 (19.28)	**9.49e-06**	Chi-square
	Low	105 (63.64)	38 (46.34)	67 (80.72)		

POLD1 expression group: The median of the POLD1 expression was cutoff value.

NMIBC: non-muscular invasive bladder cancer;

MIBC: muscular invasive bladder cancer.

Statistical significance was determined by two-tailed Chi-square or two-tailed Fisher's exact test. No adjustments were made for multiple comparisons.
