# Supplementary material for: DNA polymerase POLD1 promotes proliferation and metastasis of bladder cancer by stabilizing MYC
Source: Nat Commun. 2023 Apr 27;14:2421. doi: 10.1038/s41467-023-38160-x (PMC10140023; doi:10.1038/s41467-023-38160-x)
Supplement: Supplementary file 2 — Reporting Summary [file 41467_2023_38160_MOESM2_ESM.pdf]

nature portfolio

Corresponding author(s): Yi Zhang, Yu Xiao, Xinghuan Wang

Last updated by author(s): Mar 31, 2023

Reporting Summary

Nature Portfolio wishes to improve the reproducibility of the work that we publish. This form provides structure for consistency and transparency in reporting. For further information on Nature Portfolio policies, see our Editorial Policies and the Editorial Policy Checklist.

Statistics

For all statistical analyses, confirm that the following items are present in the figure legend, table legend, main text, or Methods section.

n/a	Confirmed	
☐	☒	The exact sample size (n) for each experimental group/condition, given as a discrete number and unit of measurement
☐	☒	A statement on whether measurements were taken from distinct samples or whether the same sample was measured repeatedly
☐	☒	The statistical test(s) used AND whether they are one- or two-sided<br>*Only common tests should be described solely by name; describe more complex techniques in the Methods section.*
☒	☐	A description of all covariates tested
☐	☒	A description of any assumptions or corrections, such as tests of normality and adjustment for multiple comparisons
☐	☒	A full description of the statistical parameters including central tendency (e.g. means) or other basic estimates (e.g. regression coefficient) AND variation (e.g. standard deviation) or associated estimates of uncertainty (e.g. confidence intervals)
☐	☒	For null hypothesis testing, the test statistic (e.g. F, t, r) with confidence intervals, effect sizes, degrees of freedom and P value noted<br>*Give P values as exact values whenever suitable.*
☒	☐	For Bayesian analysis, information on the choice of priors and Markov chain Monte Carlo settings
☒	☐	For hierarchical and complex designs, identification of the appropriate level for tests and full reporting of outcomes
☐	☒	Estimates of effect sizes (e.g. Cohen's d, Pearson's r), indicating how they were calculated

Our web collection on statistics for biologists contains articles on many of the points above.

Software and code

Policy information about availability of computer code

Data collection

The qRT-PCR data: iQ™ SYBR® Green Supermix (Bio-Rad, China);
The IP-MS data: Liquid Chromatography-Mass system (LC-MS) of Orbitrap Exploris 480 (Thermo Fisher Scientific, USA);
The RNA-seq data: illumina Novaseq™ 6000 (LC Bio Technology CO.,Ltd. Hangzhou, China);
The WB imaging: chemiluminescence and gel imager (ChemiDoc XRS, Bio-Rad, USA);
The immunofluorescence imaging: confocal laser microscope (C2+, Nikon, Japan);
The Dual-luciferase Reporter Assay data: Dual-luciferase Reporter Assay System kit (Promega, USA);
The MST assays: Monolith NT.115 instrument (NanoTemper,Germany);
The animal fluorescence detection experiment: Smallanimal in-vivo imaging system xtreme BI (Bruker, Germany).

Data analysis	All statistical analyses of the study were performed by R (version 4.1.2) or GraphPad Prism software (version 7) software;<br>All WB and immunofluorescence quantitative analysis were performed by Image J software (version 1.52);<br>All the statistical graphs and prognostic curves of this study were generated by GraphPad Prism software (version 7) or R (version 4.1.2) software;<br>The differentially expressed genes of RNA-seq were analyzed by R package "DEseq2" (version 1.34.0). The R package "clusterProfiler" (version 4.2.0) was used to perform Gene Set Enrichment Analysis (GSEA), Gene Ontology (GO), and Kyoto Encyclopedia of Genes and Genomes (KEGG) analysis;<br>All flow cytometry results were analyzed by FlowJo software (version 7.6);<br>Public ChIP-seq data were displayed by IGV software (version, 2.8.7);<br>The NanoPhotometer (Cat. #N60, Implen, Germany) to detect the quality and concentration of the extracted RNA.

For manuscripts utilizing custom algorithms or software that are central to the research but not yet described in published literature, software must be made available to editors and reviewers. We strongly encourage code deposition in a community repository (e.g. GitHub). See the Nature Portfolio guidelines for submitting code & software for further information.

Data

Policy information about availability of data

All manuscripts must include a data availability statement. This statement should provide the following information, where applicable:

- Accession codes, unique identifiers, or web links for publicly available datasets
- A description of any restrictions on data availability
- For clinical datasets or third party data, please ensure that the statement adheres to our policy

The mass spectrometry proteomics data generated in this study have been deposited in
the ProteomeXchange database under accession code PXD040806 (https://www.ebi.ac.uk/pride/archive/projects/PXD040806), the results of mass spectrometry assays generated in this study are provided in Supplementary Dataset 2. The RNA-seq data generated in this study have been deposited in the GEO database under accession code GSE200897 (https://www.ncbi.nlm.nih.gov/geo/query/acc.cgi?acc=GSE200897).
The publicly available GSE13507 (PMID:20059769) cohort data (The data included 165 samples of primary BLCA, 23 recurrent NMIBC, 58 paracancerous tissues, and 10 normal tissues. Among them, 165 cases of primary BLCA with matching clinical data were selected as the study object) used in this study are available in the GEO database under accession code GSE13507 (https://www.ncbi.nlm.nih.gov/geo/query/acc.cgi?acc=GSE13507),
The publicly available GSE32894 (PMID: 22553347) cohort data (RNA-seq data from 224 urothelial tissue samples in this cohort and matching clinical follow-up data were selected for our analysis) used in this study are available in the GEO database under accession code GSE32894 (https://www.ncbi.nlm.nih.gov/geo/query/acc.cgi?acc=GSE32894). The publicly available GSE126739 (PMID: 33298911) cohort data (these RNA-seq data contained three replicates of siNC and siMYC processing in the Jurkat cell line) used in this study are available in the GEO database under accession code GSE126739 (https://www.ncbi.nlm.nih.gov/geo/query/acc.cgi?acc=GSE126739), The publicly available GSE138295 (PMID: 32286315) cohort data (these data include the MYC and MYCN ChIP-seq data for four neuroblastoma cell lines) used in this study are available in the GEO database under accession code GSE138295 (https://www.ncbi.nlm.nih.gov/geo/query/acc.cgi?acc=GSE138295). The publicly available TCGA-BLCA cohort data (the data included 393 MIBC, 6 NMIBC, and 19 normal samples) were obtained from the UCSC Xena website (https://xenabrowser.net/). The publicly available UROMOL (PMID: 23201384) cohort data (the data included 16 MIBC, 460 NMIBC and matching clinical follow-up data) were downloaded from ArrayExpress website (https://www.ebi.ac.uk/biostudies/arrayexpress/studies/E-MTAB-4321?query=UROMOL%20#). The details of the cohorts used in our study are listed in Supplementary Table 5-9. The mutation data of Supplementary Fig. 1a used in our study were downloaded from the cBioPortal website (https://www.cbioportal.org/). The remaining data are available within the Article, Supplementary Information or Source Data file. Source data are provided as a Source Data file.

Human research participants

Policy information about studies involving human research participants and Sex and Gender in Research.

Reporting on sex and gender	Gender or gender-based analyses were not performed as the study focused on basic molecular mechanisms.
Population characteristics	Twelve patients who had undergone radical bladder cancer surgery at Department of Urology, Zhongnan Hospital of Wuhan University, were randomly selected. Data for patients'gender were not collected, and no analyses based on sex were performed.
Recruitment	The 12 pairs of human bladder cancer tissues and paracancerous tissues used in this study were obtained from the Department of Urology at Zhongnan Hospital of Wuhan University after radical bladder cancer surgery.
Ethics oversight	The study was approved by the Institutional Ethic Review Board at Zhongnan Hospital of Wuhan University (approval number: 2020102) and informed consents were obtained from all individuals.

Note that full information on the approval of the study protocol must also be provided in the manuscript.

Field-specific reporting

Please select the one below that is the best fit for your research. If you are not sure, read the appropriate sections before making your selection.

☒ Life sciences ☐ Behavioural & social sciences ☐ Ecological, evolutionary & environmental sciences

For a reference copy of the document with all sections, see nature.com/documents/nr-reporting-summary-flat.pdf

Life sciences study design

All studies must disclose on these points even when the disclosure is negative.

Sample size	Samples size for each experiment is indicated in the figures or corresponding figure legends.
Data exclusions	No samples or animals were excluded from the analyses.
Replication	The experiments were successfully repeated. Clear statements have been put into Methods section and Figure legends.
Randomization	The mice were randomly put into separate/groups cages for experiments.
Blinding	For mice studies, the experiments were performed in a blinded fashion when possible. Downstream analyses of mice samples (IHC and H&E) were performed in a blinded fashion, which means that people performing the assays were not aware of the treatment groups until the data analyses were completed.

Reporting for specific materials, systems and methods

We require information from authors about some types of materials, experimental systems and methods used in many studies. Here, indicate whether each material, system or method listed is relevant to your study. If you are not sure if a list item applies to your research, read the appropriate section before selecting a response.

Materials & experimental systems

n/a	Involved in the study	
☐	☒	Antibodies
☐	☒	Eukaryotic cell lines
☒	☐	Palaeontology and archaeology
☐	☒	Animals and other organisms
☒	☐	Clinical data
☒	☐	Dual use research of concern

Methods

n/a	Involved in the study	
☒	☐	ChIP-seq
☐	☒	Flow cytometry
☒	☐	MRI-based neuroimaging

Antibodies

Antibodies used

Target,Catalog No.,Supplier,Application/ Dilution or amount

For Western blot experiment:
Anti-GST-Tag, MY1903, MerryBio, IP/1µg WB/1:1000
Anti-His-Tag, 66005-1-Ig, Proteintech, IP/1µg WB/1:1000
Anti-HA-Tag, TA180128, Origene, IP/1µg WB/1:1000
Anti-GFP-Tag, sc-9996, Santa Cruz, IP/1µg WB/1:1000
Anti-Flag-Tag , F1804, Sigma, IP/1µg WB/1:1000
Anti-Myc-Tag , AE010, Abclonal, IP/1µg WB/1:1000
Anti-POLD1, sc-17776, Santa Cruz, WB/1:200
Anti-POLD1, ab264345, Abcam, IP/1µg WB/1:1000
Anti-MYC, 18583, Cell Signaling Technology, IP/1µg WB/1:1000
Anti-MYC-pT58, 46650, Cell Signaling Technology, WB/1:1000
Anti-MYC-pS62, 13748, Cell Signaling Technology, WB/1:1000
Anti-FBXW7, ab109617, Abcam, IP/1µg WB/1:1000
Anti-Cyclin A1+A2 , ab185619, Abcam, WB/1:1000
Anti-Cyclin B1 , 4135S, Cell Signaling Technology, WB/1:1000
Anti-Cyclin E1 , sc-377100, Santa Cruz, WB/1:200
Anti-Cyclin D1, sc-753, Santa Cruz, WB/1:200
Anti-N-cadherin, 13116S, Cell Signaling Technology, WB/1:1000
Anti-E-cadherin, 3195S, Cell Signaling Technology, WB/1:1000
Anti-Vimentin , 5741S, Cell Signaling Technology, WB/1:1000
Anti-Snail, 3879S, Cell Signaling Technology, WB/1:1000
Anti-MAX SC-8011 Santa Cruz, IP/1µg WB/1:200
Anti-Ubiquitin ab7254 Abcam, WB/1:1000
Anti-p53 10442-1-AP Proteintech, WB/1:1000
Anti-GAPDH, sc-365062, Santa Cruz, WB/1:200

For immunohistochemistry staining:

Anti-POLD1, sc-17776, Santa Cruz, 1:50
Anti-MYC, ab32072, Abcam, 1:100
Anti-Ki67, ab16667, Abcam, 1:200

Aor immunofluorescence:
Anti-POLD1, sc-17776, Santa Cruz, 1:50
Anti-MYC, 10828-1-AP, Proteintech, 1:100
Anti-MYC, sc-42, Santa Cruz, 1:200
Anti-FBXW7, ab109617, Abcam, 1:200
Anti-Cyclin E1 , sc-377100, Santa Cruz, 1:50
Anti-N-cadherin, 66219-1-Ig, Proteintech, 1:200
Anti-Ki67, ab16667, Abcam, 1:200

For ChIP assays:
Anti-GFP-Tag, ab290, Abcam, 8μg
Anti-MYC, ab32072, Abcam, 8μg

Validation

All antibodies were purchased from commercial companies, and validated by the data sheets of themanufacturer or citations listed below.
The following primary antibodies were used for Western blot experiments:
1) Anti-GST-Tag, validated with Western blot analysis of GST Tagged Protein. (http://www.merrybio.com.cn/products/gst-antibody.html);
2) Anti-His-Tag, validated with Western blot analysis of 6*His-tagged fusion protein (https://www.ptgcn.com/products/His-Tag-Antibody-66005-1-Ig.htm);
3) Anti-HA-Tag, validated with Western blot analysis of extracts from HEK293T cells were transfected with HA tagged LGALS3 cDNA for 48 hrs and lysed.
(https://cdn.origene.com/datasheet/ta180128.pdf);
4) Anti-GFP-Tag, validated with Western blot analysis of extracts from COS cells transfected with GFP-LacZ (https://www.scbt.com/p/gfp-antibody-b-2?requestFrom=search);
5) Anti-Flag-Tag, validated with Western blot analysis of Flag protein in CHO lysis solution (https://www.sigmaaldrich.cn/deepweb/assets/sigmaaldrich/product/documents/144/194/vol6_iss2_antiflag_m2.pdf);
6) Anti-Myc-Tag, validated with Western blot analysis of extracts from 293T transfected with Myc-NLK protein (https://abclonal.com.cn/catalog/AE010);
7) Anti-POLD1 (Santa Cruz), validated with Western blot analysis of extracts from HeLa and HL-60 whole cell lysates (https://www.scbt.com/p/dna-pol-delta-cat-antibody-a-9?requestFrom=search);
8) Anti-POLD1 (Abcam), validated with Western blot analysis of extracts from HeLa, HEK-293T, Jurkat, TCMK-1 and NIH/3T3 whole cell lysate (https://www.abcam.cn/pold1-antibody-ab264345.html);
9) anti-MYC, validated with Western blot analysis of extracts from SCLC-21H, Raji, KG-1a, HT-29 and etc. whole cell lysate (https://www.cellsignal.cn/products/primary-antibodies/c-myc-e5q6w-rabbit-mab/18583?site-search-type=Products&N=4294956287&Ntt=18583&fromPage=plp&_requestid=5079902);
10) anti-MYC-pT58, validated with Western blot analysis of extracts from Daudi, EL4 and Raji cells treated or not with MG132 (https://www.cellsignal.cn/products/primary-antibodies/phospho-c-myc-thr58-e4z2k-rabbit-mab/46650?site-search-type=Products&N=4294956287&Ntt=46650&fromPage=plp&_requestid=5080169);
11) Anti-MYC-pS62, validated with Western blot analysis of extracts from KARPAS-299 cells treated or not with MG132 (https://www.cellsignal.cn/products/primary-antibodies/phospho-c-myc-ser62-e1j4k-rabbit-mab/13748?site-search-type=Products&N=4294956287&Ntt=13748&fromPage=plp&_requestid=5080623);
12) anti-FBXW7, validated with Western blot analysis of extracts from HepG2, HCT 116, PANC-1, PANC-1 and MCF7 Whole Cell Lysate (https://www.abcam.cn/fbxw7-antibody-ab109617.html);
13) anti-Cyclin A1+A2, validated with Western blot analysis of extracts from HepG2 and HeLa Whole Cell Lysate (https://www.abcam.cn/cyclin-a1--cyclin-a2-antibody-epr18054-ab185619.html);
14) anti-Cyclin B1, validated with Western blot analysis of extracts from untreated or nocodazole treated HT29 cell (https://www.cellsignal.cn/products/primary-antibodies/cyclin-b1-v152-mouse-mab/4135?site-search-type=Products&N=4294956287&Ntt=4135s&fromPage=plp&_requestid=5091771);
15) anti-Cyclin E1, validated with Western blot analysis of extracts from JAR and Meg-01 whole cell lysates (https://www.scbt.com/p/cyclin-e-antibody-e-4?requestFrom=search);
16) anti-Cyclin D1, validated with Western blot analysis of extracts from MCF7 cell (PMID: 26766492)
17) anti-N-cadherin, validated with Western blot analysis of extracts from A172 and MCF7 whole cell lysates (https://www.cellsignal.cn/products/primary-antibodies/n-cadherin-d4r1h-xp-rabbit-mab/13116?site-search-type=Products&N=4294956287&Ntt=13116s&fromPage=plp&_requestid=5094005);
18) anti-E-cadherin, validated with Western blot analysis of extracts from PHPAC, MCF7, MIMCD3 and C2C12 whole cell lysates (https://www.cellsignal.cn/products/primary-antibodies/e-cadherin-24e10-rabbit-mab/3195?site-search-type=Products&N=4294956287&Ntt=3195s&fromPage=plp&_requestid=5094066);
19) anti-Vimentin, validated with Western blot analysis of extracts from HeLa, NIH/3T3, C6 and COS-7 whole cell lysates (https://www.cellsignal.cn/products/primary-antibodies/vimentin-d21h3-xp-rabbit-mab/5741?site-search-type=Products&N=4294956287&Ntt=5741s&fromPage=plp&_requestid=5094179)
20) anti-Snail, validated with Western blot analysis of extracts from HCT116, Hela, NIH/3T3, Rat2 and COS whole cell lysates (https://www.cellsignal.cn/products/primary-antibodies/snail-c15d3-rabbit-mab/3879?site-search-type=Products&N=4294956287&Ntt=3879s&fromPage=plp&_requestid=5094306);
21) anti-GAPDH, validated with Western blot analysis of extracts from HeLa, Jurkat, MCF7, A-431 and HL-60 whole cell lysates (https://www.scbt.com/p/gapdh-antibody-g-9?requestFrom=search).
The following primary antibodies were used for immunohistochemistry staining:

1) Anti-POLD1, validated with immunohistochemical analysis of paraffin-embedded human rectum tissue (https://www.scbt.com/p/dna-pol-delta-cat-antibody-a-9?requestFrom=search);
2) Anti-MYC, validated with immunohistochemical analysis of adenocarcinoma formalin fixed paraffin embedded tissue (https://www.abcam.cn/c-myc-antibody-y69-ab32072.html);
3) Anti-Ki67, validated with immunohistochemical analysis of formalin-fixed paraffin-embedded Human tonsil tissue. (https://www.abcam.cn/ki67-antibody-sp6-ab16667.html).

The following primary antibodies were used for immunofluorescence staining:
1) Anti-POLD1, validated with immunofluorescence analysis of methanol-fixed HeLa cells (https://www.scbt.com/p/dna-pol-delta-cat-antibody-a-9?requestFrom=search);
2) Anti-MYC, validated with immunofluorescence analysis of methanol-fixed U2OS cells (https://www.ptgcn.com/products/MYC-Antibody-10828-1-AP.htm);
3) Anti-FBXW7, validated with immunofluorescence analysis of methanol-fixed HeLa cells. (https://www.abcam.cn/fbxw7-antibody-ab109617.html).
4) Anti-Cyclin E1, the product specification of the antibody states that the antibody can be tested for immunofluorescence (https://datasheets.scbt.com/sc-377100.pdf);
5) Anti-N-cadherin, validated with immunofluorescence analysis of methanol-fixed mouse heart tissue (https://www.ptgcn.com/products/N-cadherin-Antibody-66219-1-Ig.htm);
6) Anti-Ki67, validated with immunofluorescence analysis of wild-type HAP1 cells Ki67 knockout HAP1 cells (https://www.abcam.cn/ki67-antibody-sp6-ab16667.html).

The following primary antibodies were used for ChIP assays:
1) Anti-MYC, validated with ChIP analysis of extracts from HeLa cells. (https://www.abcam.cn/c-myc-antibody-y69-chip-grade-ab32072.html);
2) Anti-GFP-Tag, validated with Western blot analysis and immunoprecipitation analysis of extracts from HEK293 cells. (https://www.abcam.cn/gfp-antibody-ab290.html).

Eukaryotic cell lines

Policy information about cell lines and Sex and Gender in Research

Cell line source(s)	SV-HUC-1, RT4, UM-UC-3, T24, 5637, J82, SCaBER and HEK 293T cells were kindly provided by Cell Bank of Chinese Academy of Science (Shanghai, China).
Authentication	Authentication was performed by Cell Bank, Chinese Academy of Sciences (Shanghai, China).
Mycoplasma contamination	All cell lines were tested negative for mycoplasma contamination.
Commonly misidentified lines (See ICLAC register)	None.

Animals and other research organisms

Policy information about studies involving animals; ARRIVE guidelines recommended for reporting animal research, and Sex and Gender in Research

Laboratory animals	The 4-week-old male nude mice (NOD/SCID) were purchased from Beijing HFK Bioscience Co., Ltd, China. All animals were kept under specific pathogen free (SPF) and temperature-controlled environment with 12h light/12h dark cycle, and free access to food and water.
Wild animals	No wild animals were used in this study.
Reporting on sex	The mice used in the study were all male, because the incidence of bladder cancer is much higher in men than in women (PMID: 27370177).
Field-collected samples	No field-collected samples were used in this study.
Ethics oversight	All work with mice was approved by and performed under the regulations of the Experimental Animal Welfare and Ethics Committee at Zhongnan Hospital of Wuhan University (ZN2021055).

Note that full information on the approval of the study protocol must also be provided in the manuscript.

Flow Cytometry

Plots

Confirm that:

- [x] The axis labels state the marker and fluorochrome used (e.g. CD4-FITC).
- [x] The axis scales are clearly visible. Include numbers along axes only for bottom left plot of group (a 'group' is an analysis of identical markers).
- [x] All plots are contour plots with outliers or pseudocolor plots.
- [x] A numerical value for number of cells or percentage (with statistics) is provided.

Methodology

Sample preparation	Transfected BLCA cells were harvested and washed twice with cold PBS, followed by centrifugation. The cells were then suspended using propidium iodide (PI, 100 µg/ml) and permeabilization solution in the cell cycle staining kit (CCS012, Multi sciences) and incubated in the dark for 30 min at room temperature. Finally, flow cytometry (Beckman Cytoflex) was used to detect the samples and FlowJo software (version 7.6) was used to analyze the results.
Instrument	Flow cytometry (Beckman Cytoflex)
Software	FlowJo software (version 7.6) was used to analyze the results.
Cell population abundance	No sorting was performed.
Gating strategy	The cell population was identified using FSC and SSC, this cell population gate is then placed on PI PE-A and PI PE-H plot, finally, the cell cycle curve was fitted.

- [x] Tick this box to confirm that a figure exemplifying the gating strategy is provided in the Supplementary Information.
