# Supplementary material for: DNA polymerase POLD1 promotes proliferation and metastasis of bladder cancer by stabilizing MYC
Source: Nat Commun. 2023 Apr 27;14:2421. doi: 10.1038/s41467-023-38160-x (PMC10140023; doi:10.1038/s41467-023-38160-x)
Supplement: Supplementary file 5 — Description of Additional Supplementary Files [file 41467_2023_38160_MOESM5_ESM.pdf]

File Name: Supplementary Data 1. The results of correlation analysis of POLD1 and differential expression analysis of RNA-seq.

Description: The data details the following contents: (1) Pearson correlation coefficients and p values of the top 100 TCGA-BLCA database genes significantly positively correlated with POLD1. (2) The results of RNA-seq differential expression gene analysis after POLD1 knockdown in 5637 cells.

File Name: Supplementary Data 2. IP-MS identifies POLD1 interacting proteins.

Description: The data details the following contents: (1) Results of mass spectrometry of immunoprecipitate product after IP-IgG in Flag-POLD1 overexpression 293T cells. (2) Results of mass spectrometry of immunoprecipitate product after IP-Flag in Flag-POLD1 overexpression 293T cells. 3. Results of proteins detected only in IP-Flag product.
